# Long non-coding RNAs PTENP1, GNG12-AS1, MAGI2-AS3 and MEG3 as tumor suppressors in breast cancer and their associations with clinicopathological parameters

**DOI:** 10.3233/CBM-230259

**Published:** 2024-05-28

**Authors:** Luděk Záveský, Eva Jandáková, Vít Weinberger, Luboš Minář, Milada Kohoutová, Ondřej Slanař

**Affiliations:** a First Faculty of Medicine, Institute of Biology and Medical Genetics, Charles University, Prague, Czech Republic; b General University Hospital, Prague, Czech Republic; c First Faculty of Medicine, Institute of Pharmacology, Charles University, Prague, Czech Republic; d Department of Pathology, Faculty of Medicine, Masaryk University, Brno, Czech Republic; e University Hospital Brno, Brno, Czech Republic; f Department of Obstetrics and Gynecology, Masaryk University, Brno, Czech Republic

**Keywords:** Breast cancer, GNG12-AS1, clinical outcomes, long non-coding RNAs, MAGI2-AS3, MEG3, NRSN2-AS1, PTENP1, UCA1

## Abstract

**BACKGROUND:**

Breast cancer is the most commonly occurring cancer worldwide and is the main cause of death from cancer in women. Novel biomarkers are highly warranted for this disease.

**OBJECTIVE:**

Evaluation of novel long non-coding RNAs biomarkers for breast cancer.

**METHODS:**

The study comprised the analysis of the expression of 71 candidate lncRNAs via screening, six of which (four underexpressed, two overexpressed) were validated and analyzed by qPCR in tumor tissues associated with NST breast carcinomas, compared with the benign samples and with respect to their clinicopathological characteristics.

**RESULTS:**

The results indicated the tumor suppressor roles of PTENP1, GNG12-AS1, MEG3 and MAGI2-AS3. Low levels of both PTENP1 and GNG12-AS1 were associated with worsened progression-free and overall survival rates. The reduced expression of GNG12-AS1 was linked to the advanced stage. A higher grade was associated with the lower expression of PTENP1, GNG12-AS1 and MAGI2-AS3. Reduced levels of both MEG3 and PTENP1 were linked to Ki-67 positivity. The NRSN2-AS1 and UCA1 lncRNAs were overexpressed; higher levels of UCA1 were associated with multifocality.

**CONCLUSIONS:**

The results suggest that the investigated lncRNAs may play important roles in breast cancer and comprise a potential factor that should be further evaluated in clinical studies.

## 1. Introduction

Globally, female breast cancer is the most common form of cancer and the leading cause of death from cancer in women. It recently accounted for 2.26 million new cases and 685 thousand deaths per year, ranking in first position in terms of its incidence in the vast majority of countries worldwide and 110 countries with respect to mortality [1]. The development of novel diagnostic and screening, prediction/prognosis and treatment options and tools for breast cancer patients is thus crucial in terms of improving current tumor burden and patient outcomes.

Long non-coding RNAs (lncRNAs), which comprise RNA transcripts of larger than 200 nucleotides, are thought to play important roles in the cell proliferation, differentiation, migration and apoptosis processes, which potentially contribute to the pathogenesis of many diseases, including cancer. This group of non-coding RNAs (so termed since they do not encode proteins but, according to recent findings, may encode peptides (e.g. [2])) comprises six major categories, i.e. sense lncRNAs, antisense lncRNAs, bidirectional lncRNAs, intron lncRNAs, intergenic lncRNAs and enhancer lncRNAs (see [3, 4]). LncRNAs are thought to regulate gene expression in various ways due to their involvement in both transcriptional and post-transcriptional regulation. Moreover, lncRNAs are thought to interfere with mRNA splicing and to participate in mRNA degradation and they may also affect and regulate protein stability and participate in epigenetic regulation, e.g. DNA methylation and histone modification. Concerning cancer, drug resistance may be closely related to the abnormal expression of long non-coding RNAs that act in concert with microRNAs [5]. Put simply, the functions of lncRNAs are complicated and have not yet been fully determined (see [4]). In a similar way to other non-coding RNAs (such as microRNAs), lncRNAs occur not only within cells and tissues, but also in body fluids, which makes them a useful tool in terms of their potential use as molecular biomarkers due to their altered expression (see [6]).

Over the last few years, several studies have investigated the expression of long non-coding RNAs in breast cancer using various types of samples, including body fluids (e.g. [7, 8]) and demonstrated the role of lncRNAs as potential biomarkers. Along with other studies that focused on tumor tissues and cell lines, particular lncRNAs have been identified in terms of their contribution to the development of breast cancer or their roles as tumor suppressors, and have demonstrated associations with clinical parameters including patient outcomes and therapy responses. With respect to breast tumorigenesis, the cellular functions of various lncRNAs have been shown to influence e.g. cell proliferation, invasion and metastasis and chemoresistance, or to affect epithelial-mesenchymal transition (EMT). Many target genes and microRNAs have been identified as being involved in these IncRNA regulatory effects (see [4], e.g. [9, 10]). However, due to the lack of a comprehensive evaluation of the differences in the expression of lncRNAs in differing geographical regions in the form of independent studies, their general roles, including their potential clinical impact on breast cancer patients, have not yet been fully determined.

This study investigated the expression of long-noncoding RNAs in both breast cancer tumor tissues and benign samples. Following initial screening, the expression of selected lncRNA candidates was further evaluated for validation purposes and with respect to their associations with clinicopathological and survival data, which served to indicate the importance of their roles in breast carcinogenesis.

## 2. Materials and methods

### 2.1 Patients and samples

The study comprised a single-center prospective study conducted in accordance with the Helsinki Declaration and approved by the multi-centric Ethics Committee of the General University Hospital in Prague (VFN Praha, no. 127/20 S-IV) and the Ethics Committee of the University Hospital in Brno (FN Brno). Tumor samples were obtained from patients who were receiving treatment for no-special-type (NST) invasive breast carcinoma at the University Hospital in Brno (n= 29). Benign samples (n= 29) were obtained to serve as benign counterparts of the tumors from the same patients, or were obtained via biopsies from other breast cancer patients. All the samples were obtained prior to chemotherapy or hormonal treatment. All the enrolled patients were female Caucasians. The main clinicopathological characteristics of the patients are shown in Table S1.

### 2.2 Clinical samples – pathological examination, collection and processing

The original pathological samples of the NST breast carcinomas and benign samples were examined by expert pathologists and the clinicopathological data was further reviewed by both pathologists and attending expert physicians. The pathological assessment included the determination of levels for the Ki-67 proliferation marker, human epidermal growth factor receptor 2 (Her2) and progesterone and estrogen receptors. The tumor and benign tissue samples were transferred to tubes containing an RNAlater (Thermo Fisher Scientific) liquid solution that ensured the preservation of the RNA. The samples were stored at -25^∘^C. Total RNA was isolated from the tissue samples using a mirVana miRNA Isolation Kit (Ambion/ThermoFisher Scientific, cat. no. AM1560) according to the manufacturer’s instructions, and stored at -80^∘^C.

### 2.3 Reverse transcription and qPCR

The total RNA was reverse-transcribed to cDNA using a Superscript Vilo IV kit with initial ezDNase treatment according to the manufacturer’s instructions. The cDNA thus obtained was aliquoted and stored at -25^∘^C.

The screening of the long non-coding RNA expression considered five tumor samples and five benign counterpart samples taken from the same patients, and was performed using configured 96-well plates that contained assays for 71 candidate lncRNA targets with potential roles in breast carcinogenesis and three endogenous controls (18S rRNA, GAPDH and actin beta) (TaqMan Array Human Breast Cancer lncRNA 96-well plate, standard (Configurable), Catalog number: 4391524, Thermo Fisher Scientific, Foster City, USA). Each plate served for individual samples (i.e. no pooling). The original array content was modified (some genes were removed and supplemented with other candidates based on the literature (see Table S2). The reaction volume per one target/well was 20 μl, consisting of cDNA and water (1/2 volume) and Xceed buffer (1/2 volume, IAB Czech Republic, Catalog number HPCR10502L). The qPCR amplification reactions were run on an ABI7900 device with standard thermal cycler parameters, i.e. 50^∘^C for 2 min, 95^∘^C for 10 min, 40 cycles: 95^∘^C for 15 sec, 60^∘^C for 1 min.

The validation of the expression of the candidate lncRNAs was performed employing various gene expression assays (see Table S2) that were selected according to the screening results (their differential expression and qPCR performance) and the literature. Six lncRNA genes, i.e. PTENP1, GNG12-AS1, MEG3, MAGI2-AS3, NRSN2-AS1 and UCA1 were tested for validation purposes and 18S, GAPDH and actin beta were used as endogenous controls for normalization according to geNorm analysis (qbase+). The qPCR reactions consisted of 10 μl of reaction volume per well and were run on a CFX Connect Real-Time PCR Detection System (BioRad) applying the following thermal cycler parameters: 50^∘^C for 2 min, 95^∘^C for 10 min, 40 cycles: 95^∘^C for 15 sec, 60^∘^C for 30 sec.

### 2.4 External validation of lncRNAs expression

Alterations and possible associations of lncRNA expressions with other characteristics were additionally explored and evaluated using dataset sources for breast cancer based on RNAseq data (The Cancer Genome Atlas Program (TCGA, IlluminaHiSeq) [11] and microarray expression data (Gene Expression Omnibus (GEO) repository [12, 13]. The TCGA breast cancer data sets were obtained and processed as log2(RSEM+1) data via the USSC Xena platform along with the data set for normal controls (GTEx, patients without cancer) (TCGA TARGET GTEx dataset), TCGA and TARGET Pan-Cancer dataset for invasive breast cancer samples, and GDC TCGA Breast Cancer (BRCA) for invasive ductal carcinomas, NOS [14, 15]. Data from GEO datasets were processed as normalized counts.

### 2.5 Statistical analysis

qbase+ [16, 17] and MedCalc statistical software (Belgium) were used to analyze the expression data (log-transformed CNRQ data exported from qbase+ was used in MedCalc). Two expression level cut-offs, i.e. Ct < 35 and Ct ⩽ 40 were applied in the screening and validation experiments for targets with reduced or no expression (Ct > 35). Global mean normalization was applied in the screening along with three-control normalization (18S, GAPDH and actin beta); the latter controls were also used in the validation experiment. Nonparametric Mann-Whitney tests were applied to identify differences in the expression between the pathological and control samples, and receiver operating characteristic (ROC) curve analysis was used to evaluate the area under the curve (AUC), the sensitivity and the specificity. The data used in the validation experiment was corrected for multiple testing applying the Benjamini-Hochberg procedure. The Kruskal – Wallis test, the Jonckheere-Terpstra trend test and the Mann-Whitney test were used for the testing of the associations with the stages and grades. The datasets were analyzed accordingly. P values of < 0.05 were considered significant in all the tests.

**Table 1:** Differential lncRNA expression and fold differences between breast cancer tumors and benign tissues (Screening experiment)

I. Global mean normalization				
lncRNA	FD Ct ⩽ 40	95% CI, low	95% CI, high	P
Downregulated expression				
MAGI2-AS3-Hs00416573_m1	-7.65	-18.31	-3.20	0.00794
PTENP1-Hs04272891_s1	-4.82	-10.66	-2.18	0.00794
FGF14-AS2-Hs03656456_s1	-4.53	-7.17	-2.87	0.00794
MEG3-Hs00292028_m1	-3.81	-6.74	-2.16	0.00794
MIR100HG-Hs04980371_m1	-3.34	-6.66	-1.68	0.00794
HOXA-AS2-Hs00940777_m1	-3.38	-5.88	-1.94	0.00794
Snhg14-Mm03952269_m1	-2.97	-7.10	-1.24	0.03175
Sox2ot-Mm01291217_m1	-2.97	-7.10	-1.24	0.03175
GNG12-AS1-Hs01373551_m1	-2.69	-6.03	-1.20	0.00794
LUCAT1-Hs00884761_s1	-2.01	-3.92	-1.03	0.03175
LOC100129550-Hs03644968_s1	-1.85	-2.55	-1.35	0.00794
Upregulated expression	FD Ct ⩽ 40	95% CI, low	95% CI, high	P
MIAT-Hs00978815_m1	32.70	8.51	125.67	0.00794
UCA1-Hs01909129_s1	5.68	0.65	49.50	0.03175
CDKN2B-AS1-Hs01390879_m1	4.42	1.32	14.87	0.03175
CASC2-Hs00289594_m1	2.06	1.21	3.51	0.03175
NRSN2-AS1-Hs04403463_m1	2.27	1.53	3.35	0.01587
PVT1-Hs00413039_m1	1.99	1.42	2.78	0.00794
MALAT1-Hs00273907_s1	1.72	1.06	2.80	0.03175
lncRNA	FD (Ct < 35)	95% CI, low	95% CI, high	P
Downregulated expression				
MAGI2-AS3-Hs00416573_m1	-7.02	-16.70	-2.95	0.00794
PTENP1-Hs04272891_s1	-4.42	-9.01	-2.17	0.00794
FGF14-AS2-Hs03656456_s1	-4.16	-6.52	-2.65	0.00794
HOXA-AS2-Hs00940777_m1	-3.65	-6.33	-2.10	0.01587
MEG3-Hs00292028_m1	-3.50	-6.55	-1.87	0.00794
MIR100HG-Hs04980371_m1	-3.07	-5.71	-1.65	0.00794
GNG12-AS1-Hs01373551_m1	-2.46	-5.42	-1.12	0.03175
LUCAT1-Hs00884761_s1	-1.84	-3.53	-0.96	0.03175
LOC100129550-Hs03644968_s1	-1.70	-2.33	-1.24	0.00794
Upregulated expression				
NRSN2-AS1-Hs04403463_m1	2.47	1.66	3.67	0.00794
PVT1-Hs00413039_m1	2.16	1.50	3.12	0.00794
CASC2-Hs00289594_m1	2.24	1.34	3.77	0.00794
MALAT1-Hs00273907_s1	1.88	1.25	2.83	0.03175
II. Normalization with 18S, GAPDH and actin beta				
lncRNA	FD Ct ⩽ 40	95% CI, low	95% CI, high	P
Downregulated expression				
MAGI2-AS3-Hs00416573_m1	-10.17	-25.36	-4.08	0.00794
PTENP1-Hs04272891_s1	-6.41	-15.81	-2.60	0.00794
FGF14-AS2-Hs03656456_s1	-6.02	-10.15	-3.57	0.00794
MEG3-Hs00292028_m1	-5.07	-9.14	-2.81	0.00794
HOXA-AS2-Hs00940777_m1	-4.49	-8.02	-2.51	0.00794
MIR100HG-Hs04980371_m1	-4.44	-9.08	-2.17	0.00794
Snhg14-Mm03952269_m1	-3.95	-9.81	-1.59	0.01587
Sox2ot-Mm01291217_m1	-3.95	-9.81	-1.59	0.01587
GNG12-AS1-Hs01373551_m1	-3.57	-9.41	-1.36	0.00794
LUCAT1-Hs00884761_s1	-2.66	-4.56	-1.56	0.00794
RP9P-Hs03300295_m1	-2.52	-5.24	-1.22	0.03175
LOC100129550-Hs03644968_s1	-2.46	-3.97	-1.52	0.00794
FGD5-AS1-Hs01895249_s1	-2.10	-3.69	-1.19	0.03175
PSMD6-AS2-Hs03838639_s1	-1.87	-2.79	-1.25	0.00794
SNHG6-Hs00417251_m1	-1.84	-3.26	-1.04	0.03175
GAS5-Hs05021116_g1	-1.73	-2.83	-1.06	0.03175

**Table:** 

Table 1, continued				
lncRNA	FD Ct ⩽ 40	95% CI, low	95% CI, high	P
Upregulated expression				
MIAT-Hs00978815_m1	24.61	7.56	80.12	0.00794
NRSN2-AS1-Hs04403463_m1	1.70464	1.16291	2.49873	0.03175
lncRNA	FD (Ct < 35)	95% CI, low	95% CI, high	P
Downregulated expression				
MAGI2-AS3-Hs00416573_m1	-10.17	-25.36	-4.08	0.00794
PTENP1-Hs04272891_s1	-6.41	-15.81	-2.60	0.00794
FGF14-AS2-Hs03656456_s1	-6.02	-10.15	-3.57	0.00794
HOXA-AS2-Hs00940777_m1	-5.19	-9.01	-2.99	0.01587
MEG3-Hs00292028_m1	-5.07	-9.14	-2.81	0.00794
MIR100HG-Hs04980371_m1	-4.44	-9.08	-2.17	0.00794
GNG12-AS1-Hs01373551_m1	-3.57	-9.41	-1.36	0.00794
LUCAT1-Hs00884761_s1	-2.66	-4.56	-1.56	0.00794
RP9P-Hs03300295_m1	-2.52	-5.24	-1.22	0.03175
LOC100129550-Hs03644968_s1	-2.46	-3.97	-1.52	0.00794
FGD5-AS1-Hs01895249_s1	-2.10	-3.69	-1.19	0.03175
PSMD6-AS2-Hs03838639_s1	-1.87	-2.79	-1.25	0.00794
SNHG6-Hs00417251_m1	-1.84	-3.26	-1.04	0.03175
GAS5-Hs05021116_g1	-1.73	-2.83	-1.06	0.03175
Upregulated expression				
NRSN2-AS1-Hs04403463_m1	1.71	1.16	2.50	0.03175
Consistently deregulated lncRNAs in all normalization procedures and all cut-offs				
Downregulated expression				
MAGI2-AS3-Hs00416573_m1				
PTENP1-Hs04272891_s1				
FGF14-AS2-Hs03656456_s1				
HOXA-AS2-Hs00940777_m1				
MEG3-Hs00292028_m1				
MIR100HG-Hs04980371_m1				
GNG12-AS1-Hs01373551_m1				
LUCAT1-Hs00884761_s1				
LOC100129550-Hs03644968_s1				
Upregulated expression				
NRSN2-AS1-Hs04403463_m1				

Notes: Individual lncRNA codes are as indicated in the TaqMan Assays. Two cut-offs of expression level, i.e. Ct < 35 and Ct ⩽ 40 were applied in screening experiment. FD, fold difference; CI, confidence interval. Only significant differences are noted.

**Table 2:** Differential lncRNA expression and fold differences between breast cancer tumors and benign tissues (Validation experiment)

lncRNA	FD (Ct ⩽ 40)	95% CI, low	95% CI, high	P
Downregulated expression				
PTENP1	-8.06	-12.38	-5.25	0.00000001
MAGI2-AS3	-5.89	-10.02	-3.46	0.00000020
MEG3	-3.83	-5.40	-2.72	0.00000014
GNG12-AS1	-3.35	-4.95	-2.27	0.00000141
Upregulated expression				
UCA1	2.16	1.32	3.54	0.00740687
NRSN2-AS1	1.73	1.29	2.31	0.00049781
lncRNA	FD (Ct < 35)	95% CI, low	95% CI, high	P
Downregulated expression				
PTENP1	-8.13	-12.44	-5.31	0.00000001
MAGI2-AS3	-5.89	-10.02	-3.46	0.00000020
MEG3	-3.83	-5.40	-2.72	0.00000014
GNG12-AS1	-3.35	-4.95	-2.27	0.00000141
Upregulated expression				
NRSN2-AS1	1.71	1.28	2.29	0.00060548
UCA1	1.55	1.02	2.34	0.04113247

Notes: FD, fold difference; CI, confidential interval. Three endogenous controls (18S rRNA, GAPDH and actin beta) were used for data normalizations. Two cut-offs of expression level, i.e. Ct < 35 and Ct ⩽ 40 were applied.

**Figure 1.**
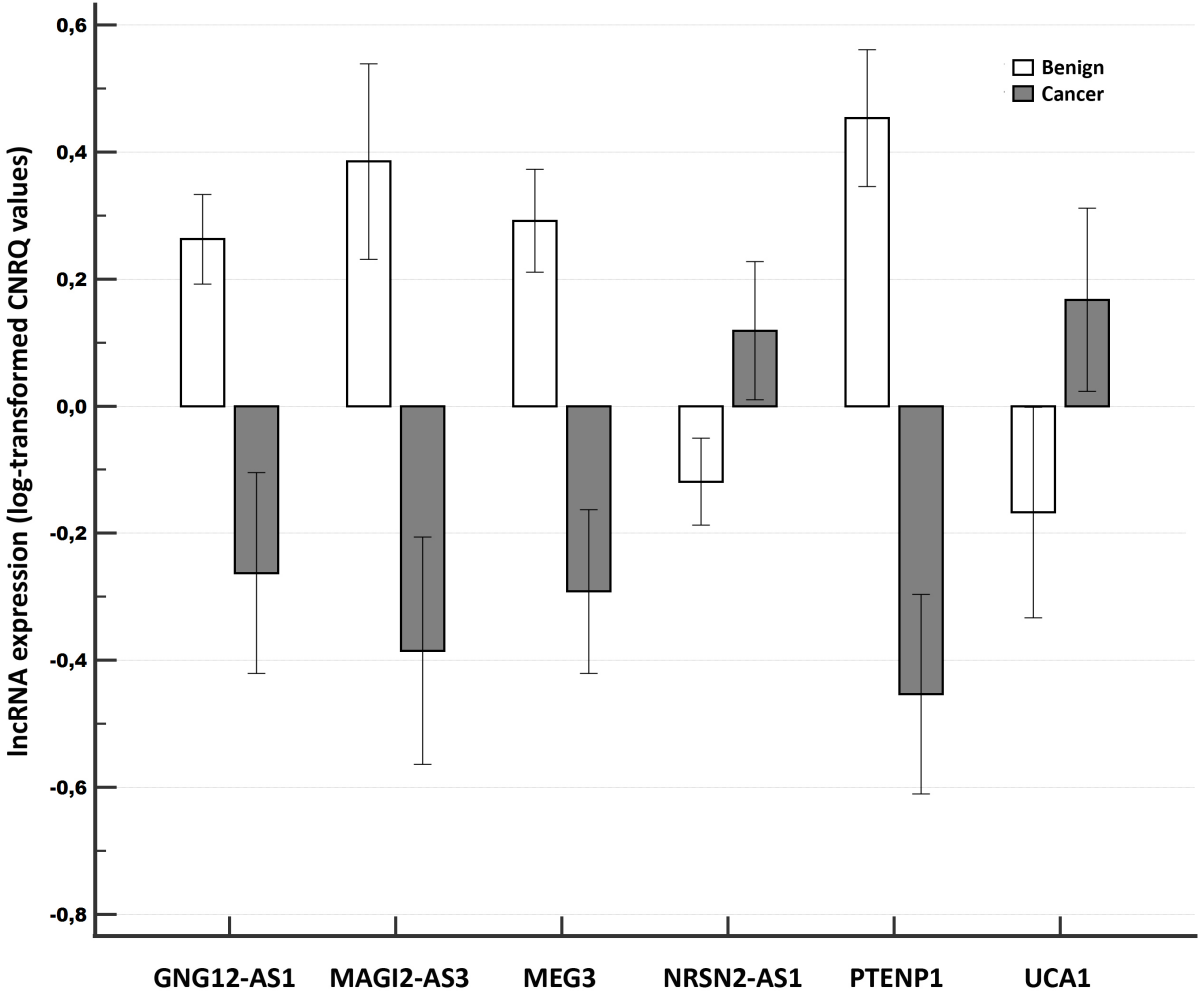
Relative expression of long non-coding RNAs compared between tumor samples of breast cancer patients and benign tissue samples in the Validation experiment. Notes: A clustered multiple-comparison graph showing mean lncRNA expression data (log-transformed CNRQ) as bar charts. Error bars indicate 95% CI for the mean.

## 3. Results

### 3.1 Screening experiment

In total, five tumor samples and five benign counterparts from the same patients were analyzed in the screening experiment. Of the 71 tested lncRNAs, and applying global mean normalization, 13 lncRNAs were found to be significantly differentially expressed (Ct < 35), of which nine were underexpressed and four were overexpressed between the cancer and the benign samples. Applying a cut-off of Ct ⩽ 40, 11 lncRNAs were observed to be underexpressed and seven overexpressed. Applying three-control normalization, 16 underexpressed lncRNAs and two overexpressed lncRNAs (Ct ⩽40), and 14 underexpressed lncRNAs along with one overexpressed lncRNA (Ct < 35) were observed. Nine lncRNAs were observed to be consistently underexpressed for all four calculation procedures, i.e. MAGI2-AS3, PTENP1, FGF14-AS2, HOXA-AS2, MEG3, MIR100HG, GNG12-AS1, LUCAT1 and LOC100129550, while only one lncRNA (NRSN2-AS1) was consistently overexpressed. For details, see Table 1.

**Figure 2.**
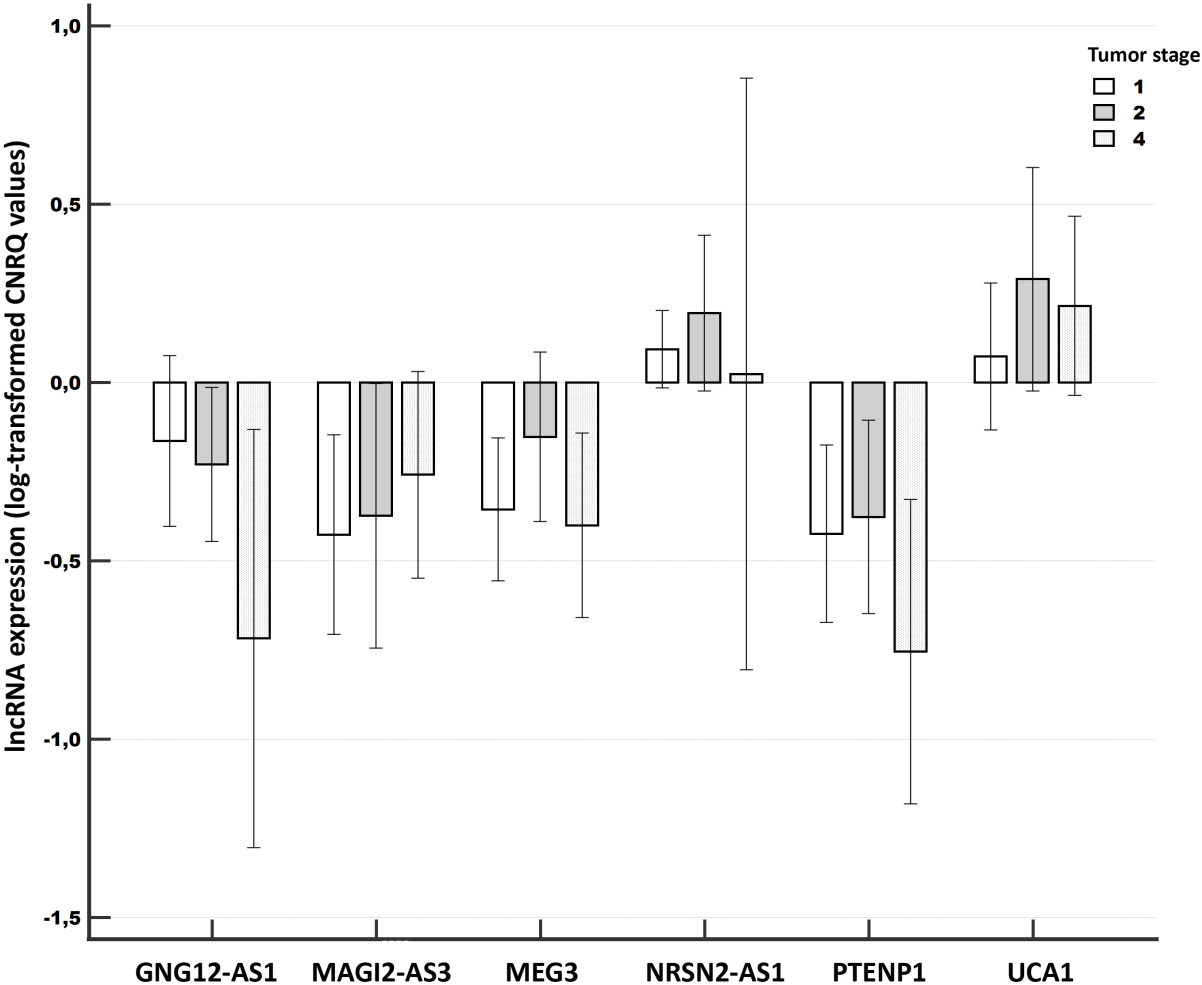
Relative expression of long non-coding RNAs compared among different tumor stages of breast cancer (Validation experiment). Notes: A clustered multiple-comparison graph showing mean lncRNA expression data (log-transformed CNRQ) as bar charts. Error bars indicate 95% CI for the mean.

**Figure 3.**
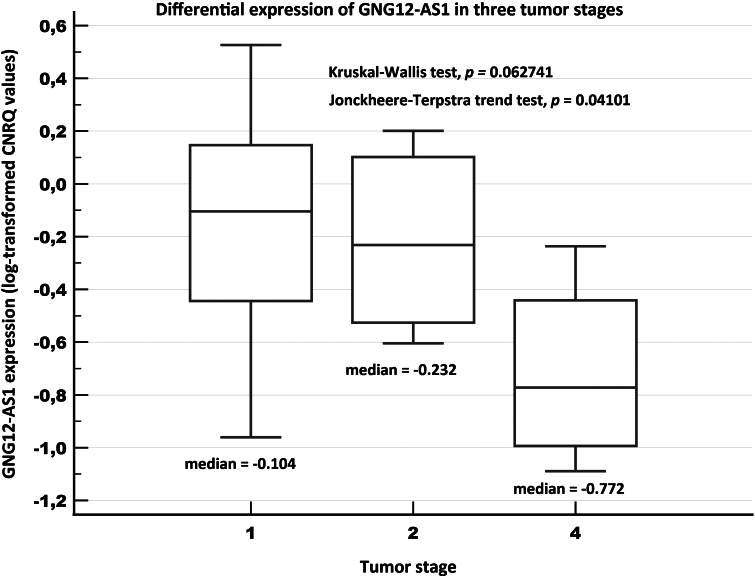
Relative expression of GNG12-AS1 in three tumor stages of breast cancer tissue samples (Validation experiment). Notes: A box of the box-plot is drawn from the 1st to 3rd quartile (the 25th and 75th percentiles). A horizontal line within a box plot represents the median. Horizontal lines are drawn at the highest value and the lowest expression value.

### 3.2 Validation experiment

The validation experiment comprised the evaluation of the altered lncRNA expression with respect to the screening experiment using 29 tumor samples and 29 benign samples. We selected downregulated PTENP1, GNG12-AS1, MEG3, MAGI2-AS3 and upregulated NRSN2-AS1 and UCA1 lncRNAs for validation purposes. The analysis of all the samples for validation reasons revealed that the six investigated lncRNAs evinced significantly differentially expressed levels, with the pattern (down or upregulation) corresponding to the screening results. The degree of downregulation of the various evaluated lncRNAs was greater (from -8.13-fold for PTENP1 to -3.35-fold for GNG12-AS1) than observed for either of the two upregulated lncRNAs (around 2-fold). For details see Table 2 and Fig. 1.

### 3.3 Receiver operating characteristics (ROC analysis)

We subsequently investigated the diagnostic power of the considered lncRNA candidate biomarkers with respect to their area under curve (AUC), sensitivity and specificity. The downregulated lncRNAs performed better than the upregulated lncRNAs.

Outstanding results were obtained for PTENP1 with AUC 0.958, with a sensitivity of 86.21% and specificity of 100%. Acceptable AUC values of ∼ 0.90 were determined for the three underexpressed lncRNAs, i.e. MEG3 (AUC 0.918, sensitivity 79.31%, specificity 100%), MAGI2-AS3 (AUC: 0.907, sensitivity 79.31%, specificity 89.66%) and GNG12-AS1 (AUC: 0.871, sensitivity 68.97%, specificity 100%). The results obtained for the overexpressed lncRNAs revealed lower values, i.e. NRSN2-AS1: AUC 0.782, sensitivity 75.86%, specificity 68.97% and UCA1: AUC 0.719, sensitivity 55.17%, specificity 89.66%. We then applied logistic regression and combined the expressions for MEG3, MAGI2-AS3 and GNG12-AS1, which resulted in the determination of outstanding values for this three-lncRNA signature panel, i.e. an AUC of 0.973, sensitivity of 89.66% and specificity of 96.55%. For details see Table S3.

### 3.4 Associations with the clinicopathological data

#### 3.4.1 Age, stage and grade

No association was determined between the expression data and the age.

Concerning the tumor stage, we determined a marginally significant association for GNG12-AS1 (p= 0.062741), which revealed a significant lower the expression the more advanced the stage trend ($p_{\text{𝑡𝑟𝑒𝑛𝑑}}=$ 0.04101). For details see Figs 2 and 3.

Concerning the clinical stage, lower levels of GNG12-AS1 were significantly associated with more advanced stages (p= 0.044762, $p_{\text{𝑡𝑟𝑒𝑛𝑑}}=$ 0.01188), revealing significant differences between clinical stage 1 and stages 3 and 4, and clinical stage 2 and clinical stage 4 (p< 0.05).

**Figure 4.**
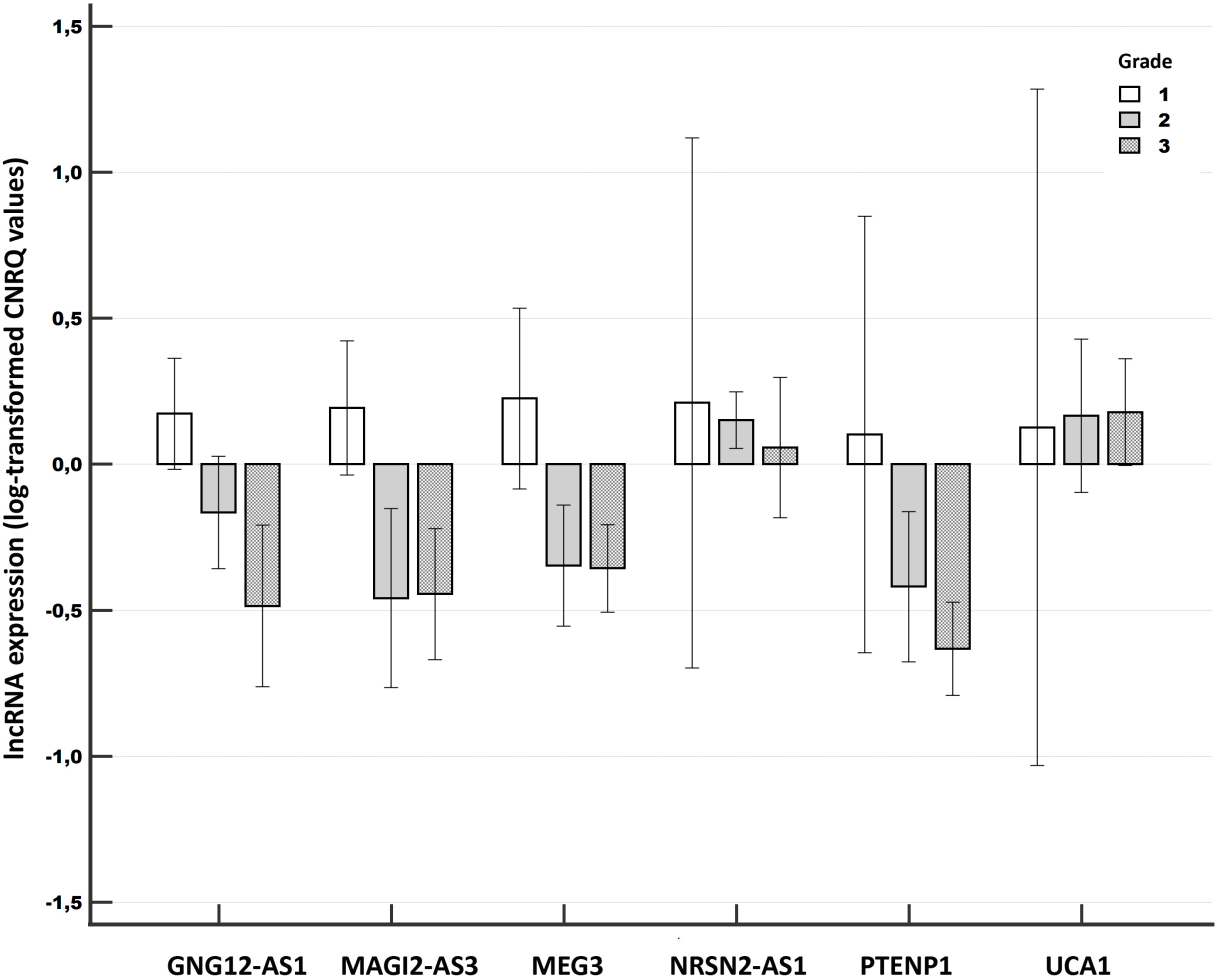
Relative expression of long non-coding RNAs compared among different tumor grades of breast cancer (Validation experiment). Notes: A clustered multiple-comparison graph showing mean lncRNA expression data (log-transformed CNRQ) as bar charts. Error bars indicate 95% CI for the mean.

We determined significant associations between the lncRNA expression and the grade for three lncRNAs. Significant differences were observed particularly between grades 1 and 3 (GNG12-AS1, p= 0.0433, PTENP1, p= 0.0094 and MAGI2-AS3, p= 0.0094), the latter also revealing a difference between grades 1 and 2 (p= 0.0322). A lower lncRNA expression was recorded for the more advanced grades in all these associations. The course of the lncRNAs expression changes in relation to the tumor grade is depicted in Fig. 4.

#### 3.4.2 Multifocality and lymph node metastasis (LNM)

A significant association between higher expression and multifocality was determined for UCA1 (p= 0.0206) (Figure S1), while the low expression of MEG3 was marginally significantly (p= 0.0672) associated with this clinical parameter. Only a marginally significant association between LNM positivity and higher expression was noted for UCA1 (p= 0.0709).

#### 3.4.3 Ki-67 proliferation marker, estrogen (ER) and progesterone (PR) receptors

Both MEG 3 and PTENP1 indicated that their lower expression was linked significantly with the Ki-67-positive samples when the two common cut-offs 20% (MEG3, p= 0.0328, Figure S2; PTENP1, p= 0.0447, Figure S3) and 15% (MEG3, p= 0.0401; PTENP1, p= 0.0132) were applied.

**Figure 5.**
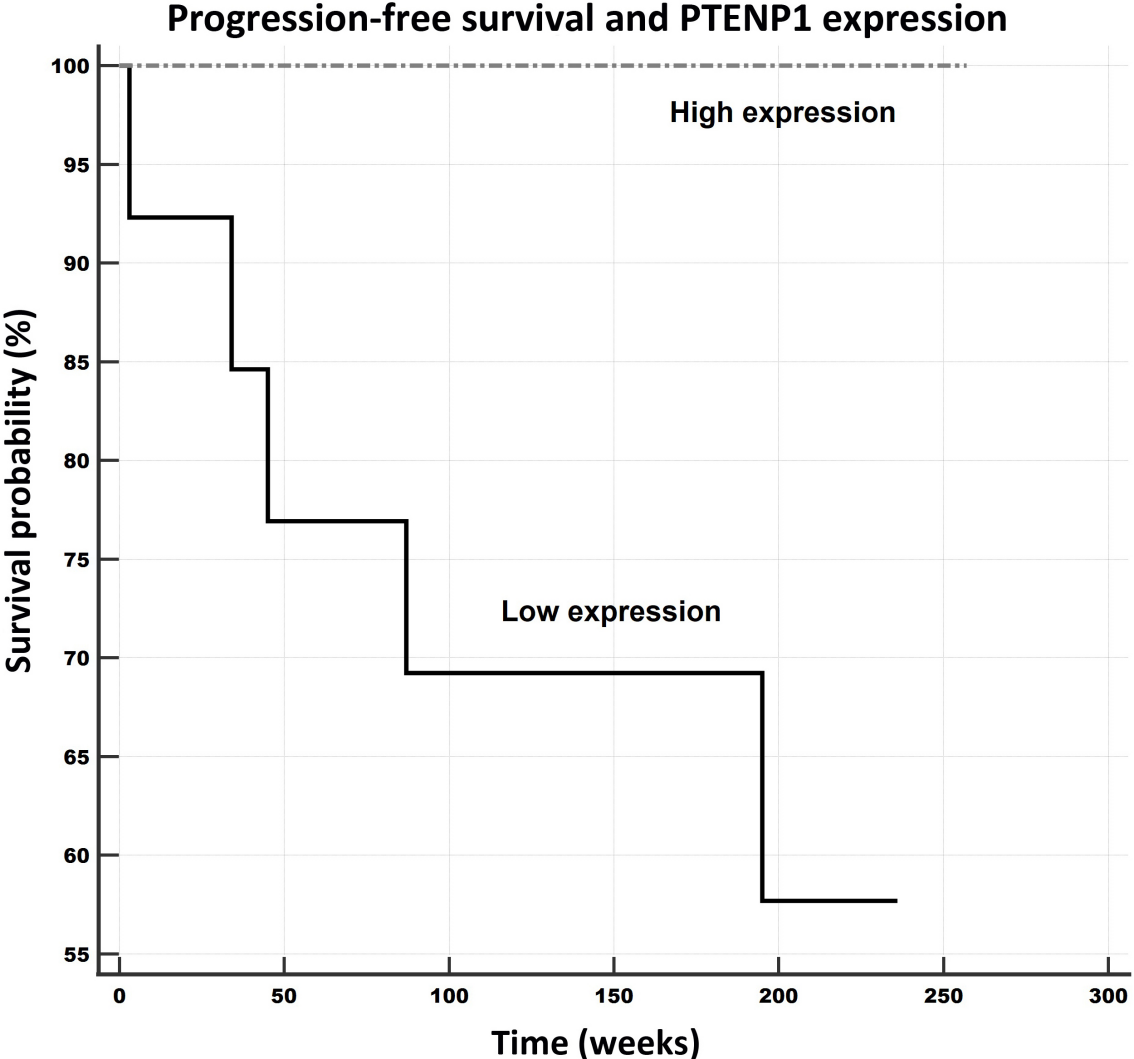
Progression-free survival in relation to PTENP1 expression levels. Notes: Univariate Kaplan-Meier survival curves for progression-free survival (PFS) related to low and high concentrations of PTENP1 in breast cancer tumors. Mean PFS for the low expression subgroup was 3.3 years (171.7 weeks), and for the high expression subgroup it was 4.9 years (257 weeks).

**Figure 6.**
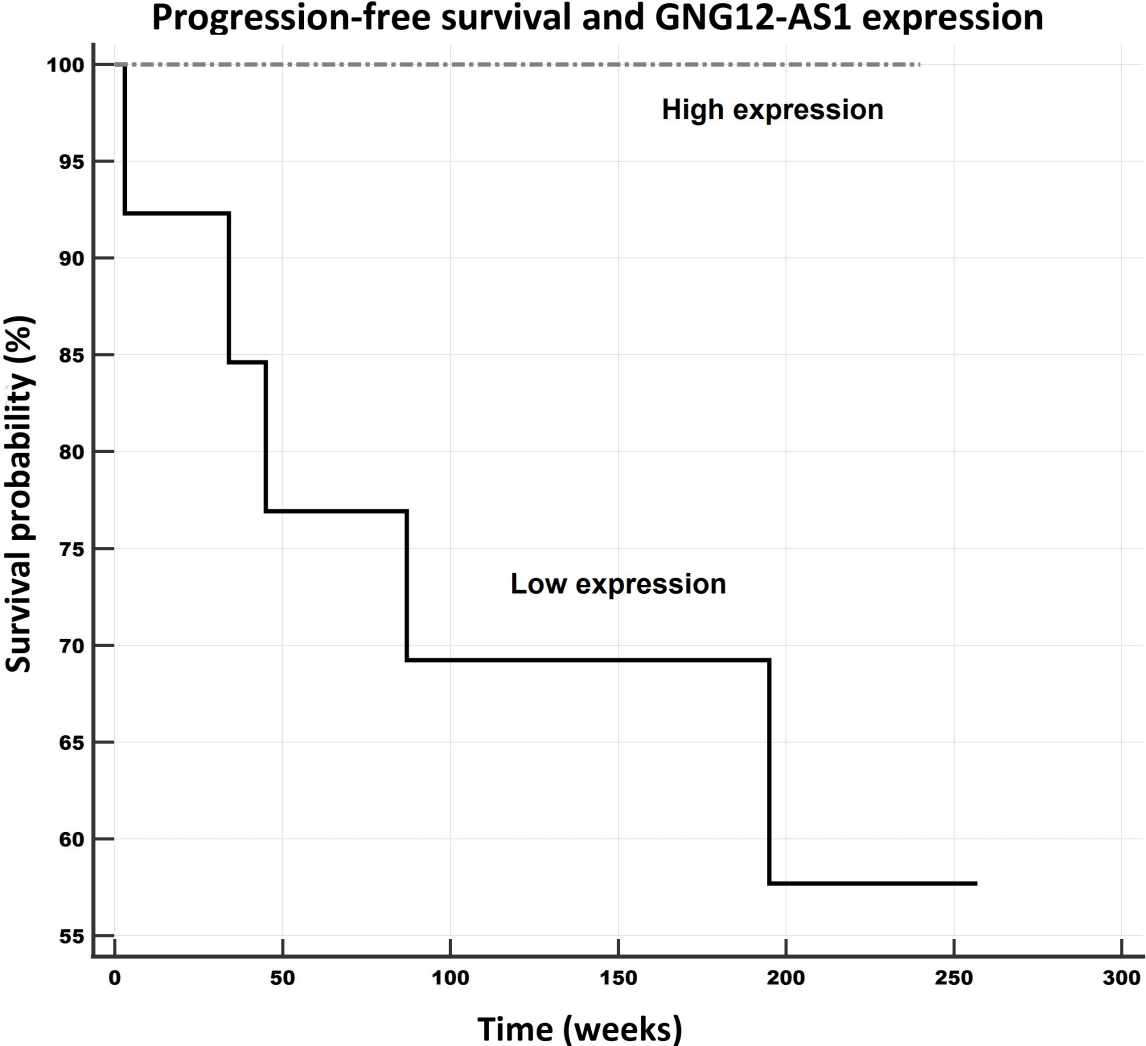
Progression-free survival in relation to GNG12-AS1 expression levels. Notes: Univariate Kaplan-Meier survival curves for progression-free survival (PFS) related to low and high concentrations of GNG12-AS1 in breast cancer tumors. Mean PFS for the low expression subgroup was 3.5 years (183.8 weeks), and for the high expression subgroup it was 4.6 years (240 weeks).

ER positivity (10% cut-off) was significantly linked with the elevated expression of NRSN2-AS1 (p= 0.0499, Figure S4), and also marginally significantly for GNG12-AS1 (p= 0.0766). A similar (but only marginally significant) pattern was observed for the dual positivity of ER and PR (10% cut-off) and the increased expression of these two lncRNAs (GNG12-AS1, p= 0.0611; NRSN2-AS1, p= 0.0738).

A marginally significant association was noted for PR negativity (10% cut-off) and the lower expression of GNG12-AS1 (p= 0.0682).

### 3.5 Survival analysis – progression-free survival, overall survival

The samples were divided into low and high expression subgroups according to the levels of the particular lncRNA expressions, and the data was analyzed with respect to the patient outcomes (progression-free survival – PFS overall survival – OS) applying Kaplan-Meier survival analysis and log-rank tests. The data on two of the patients was excluded, one due to the lack of a follow-up and the other due to fatal post-surgery complications. In total, the outcome data for 27 patients was considered in the survival analysis. The follow-up time of the patients ranged from 34 to 257 weeks (4.93 years); the median was 218 weeks (95% CI for the median: 185.6 to 225.0).

The PFS survival rate was observed to be significantly and remarkably better for the patients whose expression levels of PTENP1 (PFS: 257 weeks/59.1 months, see Fig. 5) and GNG12-AS1 (PFS: 240 weeks/ 55.2 months, see Fig. 6) were higher than those of the low expression subgroups (PTENP1, PFS: 172 weeks/39.6 months; GNG12-AS1 (PFS: 184 weeks/42.3 months). Moreover, this pattern was also observed for the OS rates with respect to these two lncRNAs, which evinced better outcomes for patients with higher expressions than the low expression subgroup (PTENP1: 257 weeks/59.1 months versus 213 weeks/49 months; GNG12-AS1: 240 weeks/55.2 months versus 216 weeks/49.7 months) although the results were only marginally significant (both p= 0.0559) (see Figures S5 and S6). The other results both for PFS and OS were not significant for the remaining lncRNAs. In addition, all the cases of progression or death were recorded in connection with the two afore-mentioned lncRNAs i.e. PTENP1 and GNG12-AS1 in the low expression subgroups. The associations between the lncRNA expression and the clinical outcomes are shown in Table S4. 

### 3.6 External evaluation using expression datasets

#### 3.6.1 TCGA-based data

Tumors versus controls

First, we used a combined TCGA GTEX data set as the primary dataset based on TCGA RNAseq data for breast cancer available for all six investigated lncRNAs. This dataset includes 1092 primary invasive breast cancer samples (including both ductal and lobular carcinomas), 7 metastatic, 113 adjacent (benign) and 179 normal tissue (from patients without cancer) samples.

**Figure 7.**
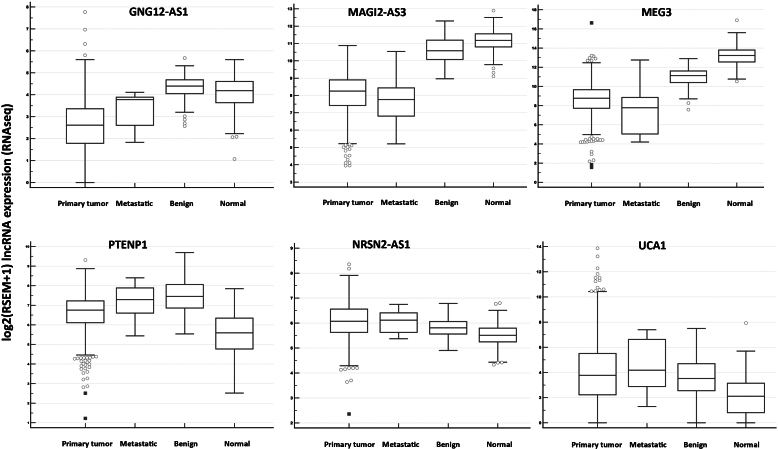
Relative expression of lncRNAs in primary tumors, metastatic samples, benign (“adjacent normal”) and normal mammary tissues in TCGA GTEX data set focused on invasive breast cancer samples.. Notes: A box of the box-plot is drawn from the 1st to 3rd quartile (the 25th and 75th percentiles). A horizontal line within a box plot represents the median. Horizontal lines are drawn at the highest value and the lowest expression value. Circles and squares represent outside values.

The expression differences in this dataset generally corresponded with our results concerning both down- and upregulations for all tested lncRNAs (see Table S5 and Fig. 7). Interestingly, PTENP1 expression was downregulated comparing primary tumors with adjacent normal tissue (similarly as in our study) but upregulated between primary tumor and normal tissue. This pattern was unique among the tested lncRNAs. However, it should be noted that this dataset contains data for all invasive breast cancer specimens.

Next, using GDC TCGA Breast Cancer (BRCA) dataset we selected invasive ductal carcinomas and compared their lncRNAs expression among four different stages groups (I-IV) available for 748 samples. Two investigated lncRNAs (MAGI2-AS3 and GNG12-AS1) evinced significant results. MAGI2-AS3 had the significant different expression between stage I and stages II, III and IV (p= 0.003178) and a significant trend to lower expression in more advanced stages ($p_{\text{𝑡𝑟𝑒𝑛𝑑}}=$ 0.01595), GNG12-AS1 showed a difference between stage I and stages II and III (p= 0.000079) with a similar trend ($p_{\text{𝑡𝑟𝑒𝑛𝑑}}=$ 0.00002). Nevertheless, no significant associations were observed in Kaplan-Meier survival analyses focused on overall survival using this data set. The lncRNA expression alterations among the different stages is presented in Figure S7.

Finally, we explored TCGA and TARGET Pan-Cancer dataset including invasive breast cancer samples using Xena hub. The expression of lncRNAs was compared according to BRCA-PAM50 characteristics (50-gene signature) of 834 samples designated as Her2, Luminal A, Luminal B, Normal and Basal subtypes [18]. As a result, significant differences were observed for all but one lncRNA (UCA1) among different subtypes (Table S5).

For example, apparently higher expression levels between normal subtype (along with luminal A) and other subtypes were demonstrated for GNG12-AS1, MEG3 and MAGI2-AS3. PTENP1 expression was lowest in the basal subtype (and luminal B) samples. NRSN2-AS1 evinced highest expression in luminal B (and luminal A) samples and the lowest in normal samples. As HER2-enriched and basal-like subtypes are considered more aggressive diseases, and luminal A should have a better prognosis than luminal B subtype, these results also supported our findings in the present study. For details see Table S6.

Cell lines

We explored data sets available for breast cancer cell lines using Xena hub (three datasets “Breast Cancer Cell Lines (Heisner 2012)”, “Cell line Encyclopedia”, “Neve 2006”). The only significant results were found in the latter data source, where we analyzed PTENP1 and MEG3 expression available (n= 57) in the Xena hub. Two data sets provided significant results, i.e. for MEG3 and Her2- positivity (lower expression in positive samples, p= 0.04547, data set 212732_at), and ER-positivity for PTENP1 (higher expression in positive samples, p= 0.01916), data set 217492_s_at).

#### 3.6.2 Geo datasets

Eight eligible Geo datasets were selected to analyze microarray expression data using breast cancer cells (GSE25976, GSE31812, GSE24460) or tissues (GSE5764, GSE23988, GSE22093, GSE3893, GSE27447). In the GSE22093 dataset, we found a significant association of grade and MEG3 expression (n= 79, lower expression in grade 3 as compared with grade 2 (p= 0.009736)). In the GSE25976 dataset, measurements of the UCA1 expression showed increased levels in metastatic variants (231BoM-1833, 231BrM-2a) of the parental cell line MDA-MB-231. The remaining datasets had only limited data as regards sample numbers and availability for the tested lncRNAs as well as a specific functional focus, therefore no significant results were obtained.

## 4. Discussion

A significant number of studies have considered the dysregulation and potential involvement of lncRNAs in breast cancer. However, the impact and roles of the various lncRNAs in breast carcinogenesis is complex and the current knowledge of these processes remains limited, which also holds true with concern to their potential contribution to patient outcomes. This study explored the potential roles of lncRNAs in breast cancer based on the expression profiles of 71 candidate lncRNAs and the detailed evaluation of six selected representative lncRNAs over a relatively large patient cohort, which revealed in particular their roles as tumor suppressors or, to a lesser extent, as oncogenes. This view is based on the mode of alterations and associations with clinicopathological data and will be discussed further.

Additional analyses of TCGA GTEX data sets provided another support for our results showing downregulated and upregulated lncRNAs in a similar pattern comparing both adjacent tissue and normal tissue controls with tumors. Interestingly, PTENP1 evinced significantly reduced expression in normal tissues as compared with tumors, metastases and adjacent benign tissues. However, the comparison of tumors versus adjacent normal tissues revealed the same mode of alteration as in our study. The downregulation of PTENP1 in normal tissues is somewhat unusual should be further investigated in detail in future. It should be noted that the dataset included all the invasive subtypes of breast cancer. 

The results for MAGI2-AS3 and GNG12-AS1 based on TCGA Breast Cancer (BRCA) dataset supported the associations with stage, the latter one comparably to our results. As noted in the Results section, also the Pan-Cancer dataset provided some support for the differences of lncRNA expression among Her2, Luminal A, Luminal B, Normal and Basal subtypes. Regarding breast cancer cell lines, due to limited data availability for the investigated lncRNAs only a partial support was observed, for example MEG3 and Her2-positivity, and MEG3 and grade; otherwise there was a lack of significant data.

### 4.1 The roles of lncRNAs of interest according to the present study and as indicated in the literature

#### 4.1.1 Potential tumor suppressor lncRNAs

The data obtained in this study from the screening and validation experiments confirmed that PTENP1, GNG12-AS1, MAGI2-AS3 and MEG3 may have, or provide support for, potential tumor suppression roles in breast cancer. Of these 4 lncRNAs, the most remarkable underexpression (minus 8-fold) was noted for PTENP1 in the comparison of the tumors with the benign samples. This lncRNA also performed well in terms of its diagnostic performance, evincing the highest values for AUC (0.958), sensitivity (86.21%) and specificity (100%). It was also possible to infer its tumor suppressor role from the association between its lower expression and the highest grade. Further, it evinced a lower expression in the Ki-67 positive samples (both cut-offs, i.e. 15% and 20%). Ki-67 is known to be a proliferation marker that worsens patient outcomes and, previously, we found it to be associated with the levels of certain microRNAs in the plasma samples of breast cancer patients [19]. Moreover, the higher expression of PTENP1 in the survival analysis was significantly associated with improved overall survival (the marginally significant difference in the OS was 10.2 months), as well as with enhanced progression-free survival (a significant difference in the PFS, i.e. 19.6 months). Several studies that focused on PTENP1 published to date provide additional support for the role of PTENP1 as a tumor suppressor in breast cancer. Using experimental cell lines and animal models, Yndestad et al. [20] investigated the effects of PTENP1 transduction on ER-positive MCF7 and T47D and ER-negative MDA-MB-231 and C3HBA breast carcinoma cells, and determined divergent effects. PTENP1 upregulation acted to decrease the PTEN gene expression and to accelerate MCF7 tumor growth *in vivo* in both ER-positive cell lines. On the other hand, the upregulation of PTENP1 resulted in an increased PTEN gene expression and reduced metastatic potential in the two ER-negative cell lines. Moreover, PTENP1 inhibited the growth rate of ER-negative C3HBA murine breast cancer xenografts [20]. Recently, Yi et al [21] analyzed MDA-MB-231 cells treated with YPB, OPB and control peptides. YPB and OPB peptides reduced breast cancer cell viability and migration, and induced apoptosis. Additionally, both peptides inhibited xenograft tumor growth in nude mice. Finally, the authors discovered that PTENP1 showed markedly reduced H3K27me3 signal. The treatment by YPB and OPB peptides resulted in PTENP1 and PTEN transcripts upregulation in MDA-MB-231 cells and also in the mouse xenograft samples. Experimental reduction of PTENP1 led to opposite effects and enhanced cell viability. The results confirmed that YPB and OPB peptides may decrease EZH2 recruitment to the promoters of target genes, such as PTENP1 and enhance anticancer activities reducing breast cancer cell proliferation and xenograft tumor formation.

Shi et al. [22] determined lower PTENP1 levels in breast cancer tissues and cell lines and its experimental overexpression in BC cell lines (MCF-7 and MDA-MB-231), increased cell survival, colony formation, migration and invasion, but decreased apoptosis. Gao et al. [23] reported underexpressed PTENP1 (and PTENP) in breast cancer tumors compared to the adjacent tissues, as well as in MDA-MB-231 and ADR resistant BC cell MCF7/ADR and T47D/ADR cell lines. The lower expression of both PTENP1 and PTEN also correlated with advanced stage and poor prognosis. Experimentally overexpressed PTENP1 in BC cell lines resulted in limited breast cancer cell viability and reduced proliferation, migration and invasion capabilities. The reduced expression of Ki-67 (as in our clinical results) was also noted; thus, all the results demonstrated the tumor suppressive role of PTENP1 in BC cells [23]. Similarly, Li et al. [24] demonstrated the lower expression of PTENP1 (and PTEN) in breast cancer tissues, as well as of PTENP1 in all the BC cell lines (BT-20, MCF-7, MDA-MB-231 and T-47D). The authors also noted the inhibition of proliferation, migration and invasion in transfected cancer cells with increased PTENP1. With respect to the first analysis of PTENP1 in human breast cancer, Yndestad et al. [25] found no association between PTENP1 and the chemotherapy or survival responses. The published data also suggests a tumor suppressor role for other cancers. PTENP1 has been observed to be downregulated in the sera of patients with gastric cancer [26] and, with the exception of carcinomas, in osteosarcoma [27].

GNG12-AS1 comprises a further lncRNA that we identified as a potential tumor suppressor due to its evincing a lower expression (around minus 3-fold) in the tumors. Several indications served to support this role, i.e. a trend toward its low expression in more advanced stages and its underexpression in the grade 3 samples; the low expression level of GNG12-AS1 was linked to both worsened overall survival (a marginally significant difference of 5.4 months) and progression-free survival (a significant difference of 12.9 months).

It has been demonstrated previously that the transcription of DIRAS3 and GNG12-AS1 is coordinately downregulated in breast cancers [28]. Functionally, GNG12-AS1 is known to be a nuclear lncRNA and is transcribed in antisense orientation to the tumor suppressor DIRAS3 [29]. Their experimental data suggested that GNG12-AS1 may have a dual function in terms of controlling cell cycle progression. The concomitant transcriptional upregulation of DIRAS3 was induced by GNG12-AS1 silencing, thus indicating transcriptional interference. Moreover, as observed in breast cancer cell lines, GNG12-AS1 has the potential to reduce cell migration after its knockdown, but independent of DIRAS3. The authors further focused on the mesenchymal epithelial transition factor (MET), which is known to regulate cell migration and invasion, and determined that GNG12-AS1 regulates MET signaling independently of DIRAS3, which indicates that GNG12-AS1 transcripts are cell migration inhibitors [29]. No other data is available that indicates or elucidates the impact of GNG12-AS1 on breast cancer.

Concerning our study, MEG3 was underexpressed (around minus 3.8-fold) in the tumors, which suggests its role as a further tumor suppressor lncRNA in breast cancer. It was found (at lower levels) to be significantly associated with the Ki-67 positive samples (both cut-offs, i.e. 15% and 20%) and marginally significantly associated with multifocality. According to the literature, this lncRNA is known for its tumor suppressor roles in breast cancer, which is consistent with our research results. It has been shown that it may act to inhibit cell proliferation and invasion in breast cancer cell lines, as well as *in vivo* tumorigenesis and angiogenesis in a nude mouse xenograft model [30]. Similar findings were reported by Zhu et al. [31], according to whom MEG3 suppressed cell proliferation and glycolysis and induced apoptosis breast cancer cells. MEG3 was further shown to be a molecular sponge for miR-21 (suppressing the expression of this oncogenic miRNA); moreover, an *in vivo* experiment also proved that overexpressed MEG3 acted to inhibit tumor growth in breast cancer by suppressing miR-21. Similarly, recent findings revealed that the knockdown of DNMT1 inhibited the progression of breast cancer cells by enhancing the MEG3 expression through demethylation [32]. Recently, Pan et al. [33] focused on similar aspects and confirmed that MEG3 was epigenetically methylated in primary breast tissues and cells, while being unmethylated in normal breast tissues and cells. As the reduced MEG3 expression resulted from the promoter methylation, inhibition of DNA methylation reversed MEG3 expression, inhibited cell proliferation and promoted cell apoptosis. MEG3 expression was negatively correlated with DNMT1 and DNMT1 knockdown increased MEG3 expression and inhibited tumor growth in mice tumor model. Overall, the results supported tumor suppressor role of MEG3 in breast cancer.

Adopting the data mining approach, Cui et al. [34] showed that DNA methylation is a factor that contributes to decreased MEG3 expression in breast cancer samples, and the positive correlation between the MEG3 expression and the estrogen receptor (ER) and the progesterone receptor (PR) status were noted. Moreover, higher levels of MEG3 were associated with enhanced overall survival (OS), relapse-free survival (RFS), distant metastasis-free survival (DMFS), and disease-specific survival (DSS) in breast cancer [34]. A similar impact on survival was reported in an earlier study [35]. Interestingly, MEG3 was seen to be highly expressed in the triple negative metastatic human Hs578T breast cancer cell line [36]. The inhibitory effects of MEG3 on cancer progression have been reported in several other reports concerning breast cancer (e.g. [37, 38, 39, 40, 41]) and other cancers such as squamous cell carcinoma of the head and neck, and lung, gastric, hepatocellular and colorectal carcinomas (see [34, 42]).

MAGI2-AS3 was found to be the second most highly underexpressed lncRNA in the tumors (minus 5.9-fold) in this study. This lncRNA was associated with the grade (its lower expression in more advanced grades). Several other reports have indicated its downregulation in breast cancer samples and its exerting of tumor-inhibitory functions support the view of this lncRNA as a tumor suppressor in breast cancer. Yang et al. [43] showed that MAGI2-AS3 significantly inhibited breast cancer cell growth and simultaneously enhanced the expression of Fas and Fas ligand (FasL). Du et al. [44] reported the inhibitory effects of MAGI2-AS3 on the migration and invasion of breast cancer cells, while its increased expression inhibited miR-374a and enhanced the expression of PTEN. Similar effects were observed by Xu et al. [45] who revealed MAGI2-AS3 as an inhibitor of cell proliferation and migration, while promoting apoptosis in MCF-7 breast cancer cells. The authors also showed that overexpressed MAGI2-AS3 is able to upregulate MAGI2 and inhibit the Wnt/β-catenin pathway, and that the DNA demethylase TET1 inhibitor is able to reverse the overexpression of MAGI2-AS3 [45]. The higher expression of MAGI2-AS3 may be associated with the enhanced relapse-free survival of triple-negative breast cancer [46]. Recently, Zhang et al. [47] demonstrated that MAGI2-AS3 encodes ORF5 polypeptide (MAGI2-AS3-ORF5) which acted as an anti-tumor peptide to hamper BRCA cell viability, proliferation, and migration. Further, MAGI2-AS3-ORF5 may interact with ECM-associated proteins and modulate breast cancer cell migration.

Other published studies on MAGI2-AS3 in various cancers revealed similar tumor suppressor functions, e.g. for non-small cell lung cancer [48], hepatocellular carcinoma [49], and glioma [50].

The opposite, i.e. oncogenic functions of MAGI2-AS3 were suggested in connection with nasopharyngeal carcinoma [51]. This study not only found that MAGI2-AS3 was elevated in NPC cell lines (especially CNE1 and SUNE1), but also determined that it promoted cell proliferation, migration and EMT processing, and even contributed to the cisplatin resistance of NPC cells, thus clearly indicating its oncogenic functions [51]. A similar report was also published for cervical squamous cell carcinoma where it was found to be overexpressed and to experimentally promote cell proliferation [52]. Both reports indicated that this lncRNA may exert divergent functions in various cancers and the associated factors should be considered in future studies.

#### 4.1.2 Potential oncogenic lncRNAs

Two lncRNAs, i.e. NRSN2-AS1 and UCA1 were included in the validation experiment as potential oncogenic lncRNAs. Both these lncRNAs evinced ambiguous results with respect to the level of upregulation (usually below 2-fold) and their diagnostic performance. Regarding the clinicopathological data, the higher expression of UCA1 was associated with multifocality and marginally significantly with lymph node metastasis. Elevated levels of NRSN2-AS1 were linked to ER positivity.

Very little research data has been published to date on the newly-discovered lncRNA NRSN2-AS1. Xu et al. [53] reported that NRSN2-AS1 was upregulated in tumor tissues (however, according to the presented graphs, the fold-differences do not appear too large) and promoted cell proliferation, migration and invasion in esophageal squamous cell carcinoma [53]. According to another study, this lncRNA was also upregulated in ovarian cancer and its knockdown contributed to the inhibition of the migration and invasion of OV cells [54]. Conversely, NRSN2-AS1 was reported to be downregulated in hepatocellular carcinoma; however, the exact level of this alteration has not been published [55]. Further research will be needed to examine the exact functions of NRSN2-AS1 for breast cancer and other cancers.

The track record of UCA1 in the literature is extensive and suggests oncogenic functions in breast cancer. For example, Tuo et al. [56] examined the interaction of UCA1 with miR-143 and found that upregulated UCA1 is able to modulate breast cancer cell growth and apoptosis via the downregulating of this miRNA. Li et al. [57] revealed that UCA1 may upregulate PTP1B and downregulate the miR-206 expression, while enhancing cell proliferation in breast cancer cells. UCA1 may also be enhanced in tamoxifen resistant LCC2 cells and their released exosomes, and the exosome-mediated transfer of UCA1 may significantly increase tamoxifen resistance in ER-positive MCF-7 cells [58]. The activity of UCA1 in tamoxifen resistance was also reported by Li et al. [59]. The enhancing effects of UCA1 were reported regarding both paclitaxel resistance [60] and doxorubicin resistance [61]. However, the impact of UCA1 on resistance to various therapeutics had already been noted in several previous investigations (see [62]).

Xiao et al. [63] experimentally applied UCA1 knockdown in the MDA-MB-231 breast cancer cell line (cells with a strong invasion capability) revealing both impaired mesenchymal properties and a reduced number of invading cells. Conversely, UCA1 upregulation enhanced the invasiveness of breast cancer cells.

Similarly, the oncogenic functions of UCA1 have also been noted in other cancers, e.g. lung cancer (e.g. [64]), cervical cancer (e.g. [65]), ovarian cancer (e.g. [66]) and a variety of other cancers (see [67]).

## 5. Conclusions

This study explored the expression of 71 candidate lncRNAs in clinical breast cancer samples compared to benign tissues and validated the expression of the best performing lncRNAs. Of the downregulated lncRNAs, PTENP1 and GNG12-AS1, in particular, evinced not only remarkably reduced expression in the cancer samples compared to the benign samples, but also showed associations with several clinical parameters including patient outcomes (improved for the high-expression subgroups), tumor grades (both lncRNAs) and stages (GNG12-AS1), thus underlining their tumor suppressor effects in breast cancer; this finding is also supported by the results of other researchers. The expression of MEG3 (along with PTENP1) was associated with the Ki-67 status (lower expression in the Ki-67-positive samples). The expression of MAGI2-AS3 was also associated with the grade. The three-lncRNA signature panel of MEG3, MAGI2-AS3 and GNG12-AS1 resulted in outstanding diagnostic parameter values (an AUC of 0.973, sensitivity of 89.66% and specificity of 96.55%). The data for the two overexpressed, potentially oncogenic lncRNAs (i.e. NRSN2-AS1 and UCA1) remains inconclusive due to the lack of significant associations, although higher levels of UCA1 were associated with multifocality.

The analyses of the TCGA Breast Cancer datasets altogether supported our results. Notably, the roles of PTENP1 remain to be further elucidated as its expression in this dataset was lower when normal tissues were compared with tumors, but it was lower also in comparison between tumors and adjacent tissues. Other datasets (TCGA GTEX, TCGA and TARGET Pan-Cancer, GDC TCGA Breast Cancer (BRCA) dataset) also provided support for the presumable roles of investigated lncRNAs in breast cancer, particularly further supported concerning the stage associations for MAGI2-AS3 and GNG12-AS1.

Additional evidence that indicates the important roles of the above-mentioned lncRNAs in breast carcinogenesis can be also found in the literature. Future studies should, therefore, focus on elucidating the detailed biological roles of these lncRNA in terms of their potential applicability in the development of novel therapeutics, as well as the further testing of their potential as novel diagnostic, predictive and prognostic biomarkers for breast cancer.

## Author contributions

Conception: LZ, EJ.

Interpretation or analysis of data: LZ, EJ, VW, LM, OS, MK.

Preparation of the manuscript: LZ.

Revision for important intellectual content: LZ, EJ, OS.

Supervision: LZ, OS, EJ.

## Supplementary data

The supplementary files are available to download from http://dx.doi.org/10.3233/CBM-230259.

## Supplementary Material

Supplementary table and Figure
