# Supplementary material for: Long non-coding RNAs PTENP1, GNG12-AS1, MAGI2-AS3 and MEG3 as tumor suppressors in breast cancer and their associations with clinicopathological parameters
Source: Cancer Biomark. 2024 May 28;40(1):61–78. doi: 10.3233/CBM-230259 (PMC11191509; doi:10.3233/CBM-230259)
Supplement: Supplementary table and Figure [file cbm-40-cbm230259-s001.docx]

**Table S1.** Clinicopathological characteristics of patients with invasive ductal carcinomas of no special type

| **Characteristics** | **Screening experiment** | **Validation experiment** |
| --- | --- | --- |
| Number of samples | Cancer samples (n = 5),  Control benign samples (n = 5) | Cancer samples (n = 29),  Control benign samples (n = 29). 16 matched cancer/benign pairs. |
| Age, years |  |  |
| Median (range) Breast cancer  Benign | 78 (67-84)  78 (67-84) | 70 (49-89)  69 (38-89) |
|  | Cancer samples | |
| Tumor stage I (total)  Ib  Ic | 1  1 | 15  1  14 |
| Tumor stage II | 2 | 10 |
| Tumor Stage IVb | 2 | 2 |
| Tumor Stage IVc | 2 | 2 |
|  |  |  |
| Clinical stage I | 0 | 13 |
| Clinical stage II | 1 | 9 |
| Clinical stage III | 3 | 4 |
| Clinical stage IV | 1 | 3 |
|  |  |  |
| Histological grade |  |  |
| G1 | 0 | 3 |
| G2 | 3 | 14 |
| G3 | 2 | 12 |
| Pathological subtypes (cut-off 10%, 15%/20% for Ki-67) |  |  |
| Ki-67 status (Cut-off 15%) |  |  |
| Negative | 2 | 13 |
| Positive | 3 | 16 |
| Ki-67 status (Cut-off 20%) |  |  |
| Negative | 2 | 15 |
| Positive | 3 | 14 |
| ER status (Cut-off 10%) |  |  |
| Negative | 0 | 4 |
| Positive | 5 | 25 |
| PR status (Cut-off 10%) |  |  |
| Negative | 1 | 11 |
| Positive | 4 | 18 |
|  |  |  |
| Her2 status |  |  |
| Negative | 5 | 28 |
| Positive | 0 | 1 |
|  |  |  |
| Multifocal disease |  |  |
| Negative | 2 | 23 |
| Positive | 3 | 6 |
|  |  |  |
| Lymph node metastasis |  |  |
| Negative | 0 | 17 |
| Positive | 4 | 8 |
| n/a | 1 | 4 |
| Follow-up weeks (median, range) (n = 27) |  | 218 (34 to 257) |
| Recurrence/Progression (other than exitus) | 1 | 2 |
| Exitus | 0 | 3 (+1 patient excluded from survival analyses*) |

Notes: ER, estrogen receptor; Her2, Receptor tyrosine-protein kinase erbB-2; Ki-67: Proliferation marker Ki-67;

PR, progesterone receptor. * post-surgery complications, n/a – not known/available

**Table S2.** List of lncRNA assays and controls used in the study

| **Assay ID** | **Gene symbol** | **Gene specification** |
| --- | --- | --- |
| Hs04967751_m1 | GACAT3 | gastric cancer associated transcript 3 (non-protein coding) |
| Hs04274319_m1 | ITGB2-AS1 | ITGB2 antisense RNA 1 |
| Hs00536197_s1 | AFAP1-AS1 | AFAP1 antisense RNA 1 |
| Hs00416024_g1 | BCAR4 | breast cancer anti-estrogen resistance 4 (non-protein coding) |
| Hs01371565_g1 | BCDIN3D-AS1 | BCDIN3D antisense RNA 1 |
| Hs04403001_s1 | CCAT2 | colon cancer associated transcript 2 (non-protein coding) |
| Hs00287056_m1 | CFLAR-AS1 | CFLAR antisense RNA 1 |
| Hs01399010_g1 | DANCR | differentiation antagonizing non-protein coding RNA |
| Hs04275636_s1 | DSCAM-AS1 | DSCAM Antisense RNA 1 |
| Hs04408008_m1 | EZR-AS1 | EZR antisense RNA 1 |
| Hs01895249_s1 | FGD5-AS1 | FGD5 antisense RNA 1 |
| **Hs01373551_m1** | **GNG12-AS1** | **GNG12 antisense RNA 1** |
| **Hs00416573_m1** | **MAGI2-AS3** | **MAGI2 antisense RNA 3** |
| Hs00399294_g1 | H19 | H19, imprinted maternally expressed transcript (non-protein coding) |
| Hs05502358_s1 | HOTAIR | HOX transcript antisense RNA |
| Hs05023182_g1 | NORAD | non-coding RNA activated by DNA damage |
| Hs03885257_s1 | GHET1 | gastric carcinoma proliferation enhancing transcript 1 |
| Hs01572486_m1 | LINC00960 | long intergenic non-protein coding RNA 960 |
| Hs01393992_g1 | MALINC1 (LINC01024) | Mitosis Associated Long Intergenic Non-Coding RNA 1 (long intergenic non-protein coding RNA 1024) |
| Hs04407061_m1 | LURAP1L-AS1 | LURAP1L antisense RNA 1 |
| Hs01396918_m1 | MAGI1-IT1 | MAGI1 intronic transcript 1 |
| Hs00255897_s1 | EPB41L4A-AS2 (EPB41L4A-DT) | EPB41L4A antisense RNA 2 (head to head) (EPB41L4A Divergent Transcript) |
| Hs00535785_s1 | RHPN1-AS1 | RHPN1 antisense RNA 1 (head to head) |
| Hs04407714_m1 | NHS-AS1 | NHS antisense RNA 1 |
| Hs04937740_s1 | NKILA | NF-kappaB interacting long non-coding RNA |
| Hs04978213_m1 | NNT-AS1 | NNT antisense RNA 1 |
| **Hs04403463_m1** | **NRSN2-AS1** | **NRSN2 antisense RNA 1** |
| Hs03838639_s1 | PSMD6-AS2 | PSMD6 antisense RNA 2 |
| Hs04275781_s1 | PTOV1-AS1 | PTOV1 antisense RNA 1 |
| Hs04274054_m1 | ST8SIA6-AS1 | ST8SIA6 antisense RNA 1 |
| Hs03656456_s1 | FGF14-AS2 | FGF14 antisense RNA 2 |
| Hs05010753_s1 | LOC100128979, newly TPM1-AS | uncharacterized LOC100128979 (newly TPM1 antisense RNA) |
| Hs05021116_g1 | GAS5 | growth arrest specific 5 (non-protein coding) |
| Hs00978815_m1 | MIAT | myocardial infarction associated transcript (non-protein coding) |
| Hs00413039_m1 | PVT1 | Pvt1 oncogene (non-protein coding) |
| Hs03677934_s1 | PDCD4-AS1 | PDCD4 antisense RNA 1 |
| **Hs01909129_s1** | **UCA1** | **urothelial cancer associated 1 (non-protein coding)** |
| Mm01291217_m1 | Sox2ot | SOX2 overlapping transcript (non-protein coding) |
| Hs00884761_s1 | LUCAT1 | lung cancer associated transcript 1 (non-protein coding) |
| Hs00417251_m1 | SNHG6 | small nucleolar RNA host gene 6 |
| Hs03644968_s1 | LOC100129550 (newly LINC02035) | uncharacterized LOC100129550 (newly long intergenic non-protein coding RNA 2035) |
| Hs01379985_m1 | ZFAS1 | ZNFX1 antisense RNA 1 |
| Hs00289594_m1 | CASC2 | cancer susceptibility candidate 2 (non-protein coding) |
| Hs04984080_m1 | LOC101927365 (newly PNISR-AS1) | uncharacterized LOC101927365(newly PNISR antisense RNA 1) |
| Hs00327058_m1 | RMST | rhabdomyosarcoma 2 associated transcript (non-protein coding) |
| Hs03296531_m1 | HOXA11-AS | HOXA11 antisense RNA |
| Hs05044154_s1 | FENDRR | FOXF1 adjacent non-coding developmental regulatory RNA |
| Hs04938212_m1 | HOST2 (CERNA-2) | human ovarian cancer-specific transcript 2 (Competing Endogenous LncRNA 2 For MicroRNA Let-7b) |
| Hs03865909_m1 | LOC101929705 (SCIRT) | uncharacterized LOC101929705 (newly stem cell inhibitory RNA transcript) |
| Hs00390315_m1 | TP73-AS1 | TP73 antisense RNA 1 |
| Hs00935566_m1 | FEZF1-AS1 | FEZF1 antisense RNA 1 |
| Hs03301064_g1 | SNHG15 | small nucleolar RNA host gene 15 |
| Hs00398296_g1 | SRA1 | steroid receptor RNA activator 1 |
| Hs00416013_m1 | STX18-AS1 | STX18 antisense RNA 1 (head to head) |
| Hs01374271_g1 | ROR1-AS1 | ROR1 antisense RNA 1 |
| Hs00940777_m1 | HOXA-AS2 | HOXA cluster antisense RNA 2 |
| Hs04980371_m1 | MIR100HG | mir-100-let-7a-2 cluster host gene (Mir-100-Let-7a-2-Mir-125b-1 Cluster Host Gene) |
| Hs01390879_m1 | CDKN2B-AS1 | CDKN2B antisense RNA 1 |
| Hs00273907_s1 | MALAT1 | metastasis associated lung adenocarcinoma transcript 1 (non-protein coding) |
| **Hs00292028_m1** | **MEG3** | **maternally expressed 3 (non-protein coding)** |
| Hs01008264_s1 | NEAT1 | nuclear paraspeckle assembly transcript 1 (non-protein coding) |
| Hs01079824_m1 | XIST | X inactive specific transcript (non-protein coding) |
| Mm03952269_m1 | Snhg14 | small nucleolar RNA host gene 14 |
| Hs03907382_m1 | LOC101929333 | uncharacterized LOC101929333 |
| Hs00327853_m1 | AFG3L1P | AFG3 like matrix AAA peptidase subunit 1, pseudogene |
| **Hs04272891_s1** | **PTENP1** | **phosphatase and tensin homolog pseudogene 1** |
| Hs01689249_s1 | SUMO1P3 | SUMO1 pseudogene 3 |
| Hs00908432_m1 | DPY19L2P2 | DPY19L2 pseudogene 2 |
| Hs01661539_s1 | OR3A4P | olfactory receptor family 3 subfamily A member 4 pseudogene |
| Hs04979523_gH | LOC643201 | centrosomal protein 192kDa pseudogene |
| Hs03300295_m1 | RP9P | retinitis pigmentosa 9 pseudogene |

| **Endogenous controls** | | |
| --- | --- | --- |
| **Assay ID** | **Gene symbol** | **Gene specification** |
| Hs99999901_s1 | 18S | Eukaryotic 18S rRNA |
| Hs01060665_g1 | ACTB | actin beta |
| Hs02786624_g1 | GAPDH | glyceraldehyde-3-phosphate dehydrogenase |

Notes: Assay ID according to TaqMan Array Human Breast Cancer lncRNA 96-well plate, standard (Configurable), Catalog number: 4391524 or individual gene expression assays (Thermo Fisher Scientific, Foster City, USA). Assays in bold were used in the validation experiment. The selection of the assays was modified with respect to literature* (some assays were removed while other assays were added according to scientific evidence).

*e.g. Zhang, T; Hu, H; Yan, G; Wu, T; Liu, S; Chen, W; Ning, Y; Lu, Z. Long Non-Coding RNA and Breast Cancer. *Technol. Cancer Res. Treat.* **2019**, 18, 1-10.

**Table S3.** Results of the receiver operating characteristics analysis for NST breast cancer patients (n = 29) and healthy controls (n = 29) with respect to lncRNA expression

| **Variable (lncRNA expression)** | **PTENP1** | **MEG3** | **MAGI2-AS3** | **GNG12-AS1** | **NRSN2-AS1** | **UCA1** | **Combined signature PTENP1/MEG3/ MAGI2-AS3** |
| --- | --- | --- | --- | --- | --- | --- | --- |
| **Area under the ROC curve (AUC)** | **0.958** | **0.918** | **0.907** | **0.871** | **0.782** | **0.719** | **0.973** |
| **Standard Error** | 0.0234 | 0.0401 | 0.0395 | 0.0474 | 0.0608 | 0.0684 | 0.0190 |
| **95% Confidence interval ^a^** | 0.871 to 0.993 | 0.816 to 0.974 | 0.802 to 0.968 | 0.757 to 0.945 | 0.655 to 0.880 | 0.586 to 0.829 | 0.892 to 0.998 |
| **z statistic** | 19.547 | 10.415 | 10.306 | 7.823 | 4.647 | 3.208 | 24.927 |
| **Significance level *p*(Area=0.5)** | <0.0001 | <0.0001 | <0.0001 | <0.0001 | <0.0001 | 0.0013 | <0.0001 |
|  |  |  |  |  |  |  |  |
| **Youden index J** | 0.8621 | 0.7931 | 0.6897 | 0.6897 | 0.4483 | 0.4483 | 0.8621 |
| **95% Confidence interval *^a^*** | 0.6897 to 0.9655 | 0.6129 to 0.8966 | 0.4828 to 0.7931 | 0.5172 to 0.8276 | 0.2069 to 0.5862 | 0.2069 to 0.6207 | 0.7146 to 0.9655 |
| **Associated criterion** | ≤-0.04923 | ≤-0.09077 | ≤-0.04113 | ≤-0.06815 | >-0.04272 | >0.186 | >0.515462245 |
| **95% Confidence interval** | ≤-0.08727 to ≤0.2876 | ≤-0.1729 to ≤0.1343 | ≤-0.1451 to ≤0.281 | ≤-0.1041 to ≤0.2411 | >-0.2542 to >0.1244 | >0.1308 to >0.186 | >0.122051715 to >0.542210367 |
| **Sensitivity (%)** | **86.21** | **79.31** | **79.31** | **68.97** | **75.86** | **55.17** | **89.66** |
| **Specificity (%)** | **100.00** | **100.00** | **89.66** | **100.00** | **68.97** | **89.66** | **96.55** |

Notes: ROC curve analysis was performed to assess diagnostic performance of particular lncRNAs (samples of breast cancer tissues versus benign samples). Total sample size: 58. Positive group (cancer): 29 (50.0%), negative group (benign): 29 (50.0%). Statistic values from the MedCalc analysis. *a* BCa bootstrap confidence interval (1000 iterations; random number seed: 978). If *p* value is small (*p* <0.05) then the Area under the ROC curve is significantly different from 0.5 and there is evidence that the laboratory test does have an ability to distinguish between the two groups. The criterion values with a comparison sign, > or <, depending on whether higher values indicate disease, of lower values indicate disease (MedCalc).

**Table S4. Progression-free survival (PFS) and overall survival (OS) associated with lncRNA expression levels**

1. **Progression-free survival (PFS) associated with lncRNA expression levels**

| **Breast cancer patients** | **Mean PFS as calculated in Kaplan-Meier tests (weeks)** | **SE** | **95% CI for the mean** | **Endpoints for PFS** | **Log-rank test for PFS** |
| --- | --- | --- | --- | --- | --- |
| **lncRNA (Expression level)** |  |  |  |  |  |
| PTENP1 – low expression | 171.654 | 24.750 | 123.144 to 220.163 | 5 | *p* = 0.0098  HR: n/a |
| PTENP1 – high expression | 257.000 | 0.000 | 257.000 to 257.000 | 0 |  |
|  | | | | | |
| GNG12-AS1– low expression | 183.769 | 27.364 | 130.137 to 237.402 | 5 | *p* = 0.0098  HR: n/a |
| GNG12-AS1– high expression | 240.000 | 0.000 | 240.000 to 240.000 | 0 |  |
|  | | | | | |
| MEG3 – low expression | 205.373 | 23.846 | 158.634 to 252.112 | 4 | *p* = 0.1877*  HR for high expression: 0.3075 [0.05322 to 1.7772] |
| MEG3 – high expression | 215.769 | 13.672 | 188.971 to 242.567 | 1 |  |
|  | | | | | |
| MAGI2-AS3 – low expression | 228.929 | 18.571 | 192.529 to 265.328 | 2 | *p* = 0.6336*  HR for high expression: 1.5347 [0.2637 to 8.9328] |
| MAGI2-AS3 – high expression | 202.962 | 21.592 | 160.642 to 245.281 | 3 |  |
|  | | | | | |
| NRSN2-AS1– low expression | 207.429 | 15.576 | 176.899 to 237.958 | 2 | *p* = 0.5723*  HR for high expression: 1.6597  [0.2859 to 9.6347] |
| NRSN2-AS1– high expression | 214.479 | 23.879 | 167.675 to 261.282 | 3 |  |
|  |  |  |  |  |  |
| UCA1– low expression | 210.929 | 16.630 | 178.334 to 243.523 | 2 | *p* = 0.6336*  HR for high expression: 1.5347 [0.2637 to 8.9328] |
| UCA1– high expression | 215.908 | 23.347 | 170.148 to 261.667 | 3 |  |

**B. Overall survival (OS) associated with lncRNA expression levels**

| **Breast cancer patients** | **Mean OS as calculated in Kaplan-Meier tests (weeks)** | **SE** | **95% CI for the mean** | **Endpoints for OS** | **Log-rank test for OS** |
| --- | --- | --- | --- | --- | --- |
| **lncRNA (Expression level)** |  |  |  |  |  |
| PTENP1 – low expression | 212.780 | 21.373 | 170.888 to 254.672 | 3 | *p* = 0.0559**  HR: n/a |
| PTENP1 – high expression | 257.000 | 0.000 | 257.000 to 257.000 | 0 |  |
|  | | | | | |
| GNG12-AS1– low expression | 216.407 | 21.947 | 173.391 to 259.422 | 3 | *p* = 0.0559**  HR: n/a |
| GNG12-AS1– high expression | 240.000 | 0.000 | 240.000 to 240.000 | 0 |  |
|  | | | | | |
| MEG3 – low expression | 235.593 | 15.821 | 204.583 to 266.602 | 2 | *p* = 0.6480*  HR for high expression: 0.5886 [0.06047 to 5.7288] |
| MEG3 – high expression | 215.769 | 13.672 | 188.971 to 242.567 | 1 |  |
|  | | | | | |
| MAGI2-AS3 – low expression | 241.071 | 15.349 | 210.987 to 271.156 | 1 | *p* = 0.6174*  HR for high expression: 1.7860 [0.1835 to 17.3844] |
| MAGI2-AS3 – high expression | 231.092 | 15.632 | 200.454 to 261.731 | 2 |  |
|  | | | | | |
| NRSN2-AS1– low expression | 217.714 | 12.802 | 192.622 to 242.807 | 1 | *p* = 0.5859*  HR for high expression: 1.8834 [0.1931 to 18.3743] |
| NRSN2-AS1– high expression | 234.400 | 16.856 | 201.362 to 267.438 | 2 |  |
|  |  |  |  |  |  |
| UCA1– low expression | 221.571 | 13.904 | 194.320 to 248.823 | 1 | *p* = 0.6633*  HR for high expression: 1.6657 [0.1675 to 16.5657] |
| UCA1– high expression | 235.741 | 15.984 | 204.412 to 267.071 | 2 |  |

Notes: The mean survival time was calculated in MedCalc as the area under the survival curve in the interval 0 to *t*_max_ (Klein & Moeschberger, 2003). * Non-significant results. **Marginally significant results. Reference: Klein JP, Moeschberger ML (2003) Survival Analysis. Techniques for censored and truncated data, 2^nd^ ed. New York: Springer Publishers. HR = hazard ratio with 95% confidence interval in square brackets, n/a = not available. HR could not be calculated in case when only one type of events was available.

**Table S5. Differential lncRNA expression in primary tumors, metastatic tissues, adjacent normal (benign) samples and normal tissues (TCGA GTEX Breast cancer dataset, RNASeq data)**

| **Sample type** | **LncRNA** | **n** | **Minimum** | **25th percentile** | **Median** | **75th percentile** | **Maximum** | **Kruskal-Wallis test (Different (P<0,05))** | **Jonckheere-Terpstra trend test (*p*)** |
| --- | --- | --- | --- | --- | --- | --- | --- | --- | --- |
|  |  |  | **log2(RSEM+1) expression** | | | | |  |  |
| 1) Primary tumor (PT) | **PTENP1** | 1092 | 1.223 | 6.109 | **6.753** | 7.224 | 9.306 | (3)(4) | <0.00001 |
| 2) Metastatic |  | 7 | 5.438 | 6.601 | **7.289** | 7.885 | 8.401 | (4) |  |
| 3) Adjacent normal tissue (ANT) |  | 113 | 5.537 | 6.859 | **7.454** | 8.059 | 9.69 | (1)(4) |  |
| 4) Normal tissue (NT) |  | 179 | 2.516 | 4.768 | **5.592** | 6.344 | 7.846 | (1)(2)(3) |  |
| log2-FD PT versus ANT |  |  |  |  | **-0.701** |  |  |  |  |
| log2-FD PT versus NT |  |  |  |  | **1.161** |  |  |  |  |
|  |  |  |  |  |  |  |  |  |  |
| 1) Primary tumor | **GNG12-AS1** | 1092 | 0 | 1.788 | **2.616** | 3.362 | 7.768 | (2)(3)(4) | <0.00001 |
| 2) Metastatic |  | 7 | 1.831 | 2.608 | **3.779** | 3.89 | 4.111 | (1)(3) |  |
| 3) Adjacent normal tissue |  | 113 | 2.568 | 4.051 | **4.395** | 4.681 | 5.669 | (1)(2)(4) |  |
| 4) Normal tissue |  | 179 | 1.072 | 3.639 | **4.186** | 4.607 | 5.6 | (1)(3) |  |
| log2-FD PT versus ANT |  |  |  |  | **-1.779** |  |  |  |  |
| log2-FD PT versus NT |  |  |  |  | **-1.57** |  |  |  |  |
|  |  |  |  |  |  |  |  |  |  |
| 1) Primary tumor | **MAGI2-AS3** | 1092 | 3.95 | 7.421 | **8.253** | 8.898 | 10.88 | (3)(4) | <0.00001 |
| 2) Metastatic |  | 7 | 5.207 | 6.809 | **7.765** | 8.437 | 10.54 | (3)(4) |  |
| 3) Adjacent normal tissue |  | 113 | 8.963 | 10.075 | **10.58** | 11.192 | 12.3 | (1)(2)(4) |  |
| 4) Normal tissue |  | 179 | 9.108 | 10.803 | **11.18** | 11.558 | 12.9 | (1)(2)(3) |  |
| log2-FD PT versus ANT |  |  |  |  | **-2.327** |  |  |  |  |
| log2-FD PT versus NT |  |  |  |  | **-2.927** |  |  |  |  |
|  |  |  |  |  |  |  |  |  |  |
| 1) Primary tumor | **MEG3** | 1092 | 1.547 | 7.724 | **8.771** | 9.659 | 16.63 | (3)(4) | <0.00001 |
| 2) Metastatic |  | 7 | 4.202 | 5.04 | **7.775** | 8.851 | 12.76 | (3)(4) |  |
| 3) Adjacent normal tissue |  | 113 | 7.582 | 10.405 | **11.13** | 11.612 | 12.9 | (1)(2)(4) |  |
| 4) Normal tissue |  | 179 | 10.53 | 12.555 | **13.23** | 13.797 | 16.9 | (1)(2)(3) |  |
| log2-FD PT versus ANT |  |  |  |  | **-2.359** |  |  |  |  |
| log2-FD PT versus NT |  |  |  |  | **-4.459** |  |  |  |  |
|  |  |  |  |  |  |  |  |  |  |
| 1) Primary tumor | **NRSN2-AS1** | 1092 | 2.357 | 5.627 | **6.072** | 6.559 | 8.352 | (3)(4) | <0.00001 |
| 2) Metastatic |  | 7 | 5.372 | 5.626 | **6.116** | 6.409 | 6.748 | (4) |  |
| 3) Adjacent normal tissue |  | 113 | 4.903 | 5.561 | **5.808** | 6.06 | 6.786 | (1)(4) |  |
| 4) Normal tissue |  | 179 | 4.331 | 5.246 | **5.51** | 5.796 | 6.8 | (1)(2)(3) |  |
| log2-FD PT versus ANT |  |  |  |  | **0.264** |  |  |  |  |
| log2-FD PT versus NT |  |  |  |  | **0.562** |  |  |  |  |
|  |  |  |  |  |  |  |  |  |  |
| 1) Primary tumor | **UCA1** | 1092 | 0 | 2.226 | 3.773 | 5.511 | 13.87 | (4) | <0.00001 |
| 2) Metastatic |  | 7 | 1.293 | 2.883 | 4.181 | 6.625 | 7.395 | (4) |  |
| 3) Adjacent normal tissue |  | 113 | 0 | 2.551 | 3.523 | 4.695 | 7.496 | (4) |  |
| 4) Normal tissue |  | 178 | 0 | 0.805 | 2.111 | 3.146 | 7.931 | (1)(2)(3) |  |
| log2-FD PT versus ANT |  |  |  |  | **0.25** |  |  |  |  |
| log2-FD PT versus NT |  |  |  |  | **1.662** |  |  |  |  |

Note: The TCGA TARGET GTEx dataset was used for the analysis using Xena platform [15]. Cancer samples represent invasive breast carcinomas. Data were processed in MedCalc statistical software. FD – fold difference.

**Table S6. Differential lncRNA expression (log2(RSEM+1)) among different subtypes of breast cancer (TCGA and TARGET Pan-Cancer dataset)**

| **PTENP1** |  |  |  |  |  |  |  |
| --- | --- | --- | --- | --- | --- | --- | --- |
| **Factor** | **n** | **Minimum** | **25th percentile** | **Median** | **75th percentile** | **Maximum** | **Different (*P*<0.05) from factor no.** |
| **(1) BASAL** | 137 | 1.223 | 5.975 | 6.717 | 7.407 | 8.87 | (2)(5) |
| **(2) HER2** | 66 | 3.771 | 6.726 | 7.253 | 7.574 | 8.772 | (1)(3)(4) |
| **(3) LUMA** | 417 | 4.462 | 6.393 | 6.894 | 7.267 | 8.841 | (2) |
| **(4) LUMB** | 192 | 2.507 | 6.242 | 6.82 | 7.278 | 8.802 | (2)(5) |
| **(5) NORMAL** | 22 | 5.285 | 6.612 | 7.291 | 7.48 | 7.929 | (1)(4) |
|  |  |  |  |  |  |  |  |
| **GNG12-AS1** |  |  |  |  |  |  |  |
| **Factor** | **n** | **Minimum** | **25th percentile** | **Median** | **75th percentile** | **Maximum** | **Different (*P*<0.05) from factor no.** |
| **(1) BASAL** | 137 | 0 | 1.265 | 2.086 | 2.802 | 7.768 | (3)(5) |
| **(2) HER2** | 66 | 0 | 1.528 | 2.125 | 2.802 | 5.465 | (3)(5) |
| **(3) LUMA** | 417 | 0 | 2.543 | 3.135 | 3.692 | 5.601 | (1)(2)(4) |
| **(4) LUMB** | 192 | 0 | 1.25 | 2.082 | 2.856 | 4.585 | (3)(5) |
| **(5) NORMAL** | 22 | 1.412 | 2.53 | 3.369 | 4.034 | 4.698 | (1)(2)(4) |
|  |  |  |  |  |  |  |  |
| **MAGI2-AS3** |  |  |  |  |  |  |  |
| **Factor** | **n** | **Minimum** | **25th percentile** | **Median** | **75th percentile** | **Maximum** | **Different (*P*<0.05) from factor no.** |
| **(1) BASAL** | 137 | 4.103 | 6.953 | 7.785 | 8.51 | 10.77 | (3)(5) |
| **(2) HER2** | 66 | 5.969 | 7.457 | 8.041 | 8.469 | 9.953 | (3)(5) |
| **(3) LUMA** | 417 | 4.109 | 7.924 | 8.601 | 9.103 | 10.7 | (1)(2)(4)(5) |
| **(4) LUMB** | 192 | 3.954 | 7.235 | 7.792 | 8.462 | 10.53 | (3)(5) |
| **(5) NORMAL** | 22 | 8.169 | 8.909 | 9.357 | 9.927 | 10.5 | (1)(2)(3)(4) |
|  |  |  |  |  |  |  |  |
| **MEG3** |  |  |  |  |  |  |  |
| **Factor** | **n** | **Minimum** | **25th percentile** | **Median** | **75th percentile** | **Maximum** | **Different (*P*<0.05) from factor no.** |
| **(1) BASAL** | 137 | 2.183 | 6.45 | 7.377 | 8.299 | 16.63 | (2)(3)(4)(5) |
| **(2) HER2** | 66 | 6.316 | 7.946 | 8.653 | 9.152 | 11.91 | (1)(4)(5) |
| **(3) LUMA** | 417 | 1.848 | 8.049 | 8.931 | 9.658 | 12.7 | (1)(4)(5) |
| **(4) LUMB** | 192 | 4.203 | 7.43 | 8.239 | 8.995 | 10.76 | (1)(2)(3)(5) |
| **(5) NORMAL** | 22 | 7.47 | 9.12 | 9.692 | 10.7 | 11.39 | (1)(2)(3)(4) |
|  |  |  |  |  |  |  |  |
| **NRSN2-AS1** |  |  |  |  |  |  |  |
| **Factor** | **n** | **Minimum** | **25th percentile** | **Median** | **75th percentile** | **Maximum** | **Different (*P*<0.05) from factor no.** |
| **(1) BASAL** | 137 | 2.357 | 5.553 | 5.979 | 6.694 | 8.179 | (4)(5) |
| **(2) HER2** | 66 | 3.639 | 5.357 | 6.113 | 6.558 | 7.908 | (3)(4)(5) |
| **(3) LUMA** | 417 | 4.13 | 5.792 | 6.162 | 6.549 | 8.352 | (2)(5) |
| **(4) LUMB** | 192 | 4.352 | 5.67 | 6.297 | 6.8 | 7.717 | (1)(2)(5) |
| **(5) NORMAL** | 22 | 4.785 | 5.181 | 5.616 | 6.145 | 6.847 | (1)(2)(3)(4) |
|  |  |  |  |  |  |  |  |
| **UCA1** |  |  |  |  |  |  |  |
| **Factor** | **n** | **Minimum** | **25th percentile** | **Median** | **75th percentile** | **Maximum** | **Kruskal-Wallis test** |
| **BASAL** | 137 | -9.966 | -5.012 | -2.245 | 0.357 | 8.739 | *P* = 0.125088 |
| **HER2** | 66 | -9.966 | -4.035 | -1.688 | 0.633 | 4.695 |  |
| **LUMA** | 417 | -9.966 | -3.458 | -2.053 | -0.636 | 3.954 |  |
| **LUMB** | 192 | -9.966 | -4.035 | -2.146 | -0.534 | 5.246 |  |
| **NORMAL** | 22 | -9.966 | -2.114 | -0.771 | 0.24 | 7.505 |  |

Notes: The TCGA and TARGET Pan-Cancer dataset was used for the analysis using Xena platform [15]. Cancer samples represent invasive breast carcinomas. Data were processed in MedCalc statistical software.

**
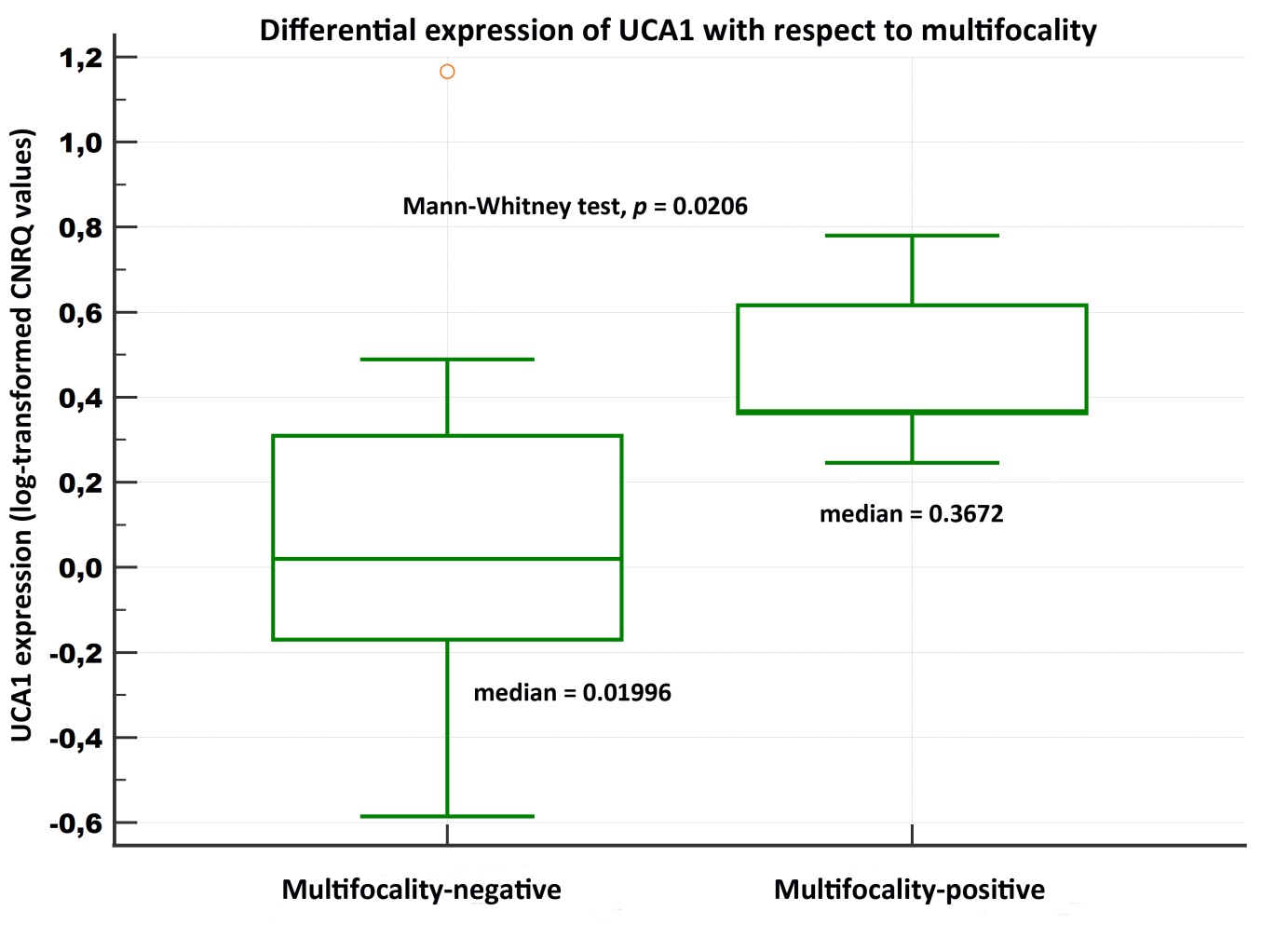
**

**Figure S1.** Relative expression of UCA1 in breast cancer tissue samples with respect to a presence of multifocal disease (validation experiment)

Notes: A box of the box-plot is drawn from the 1st to 3rd quartile (the 25th and 75th percentiles). A horizontal line within a box plot represents the median. Horizontal lines are drawn at the highest value and the lowest expression value. A circle represents an outside value.

**
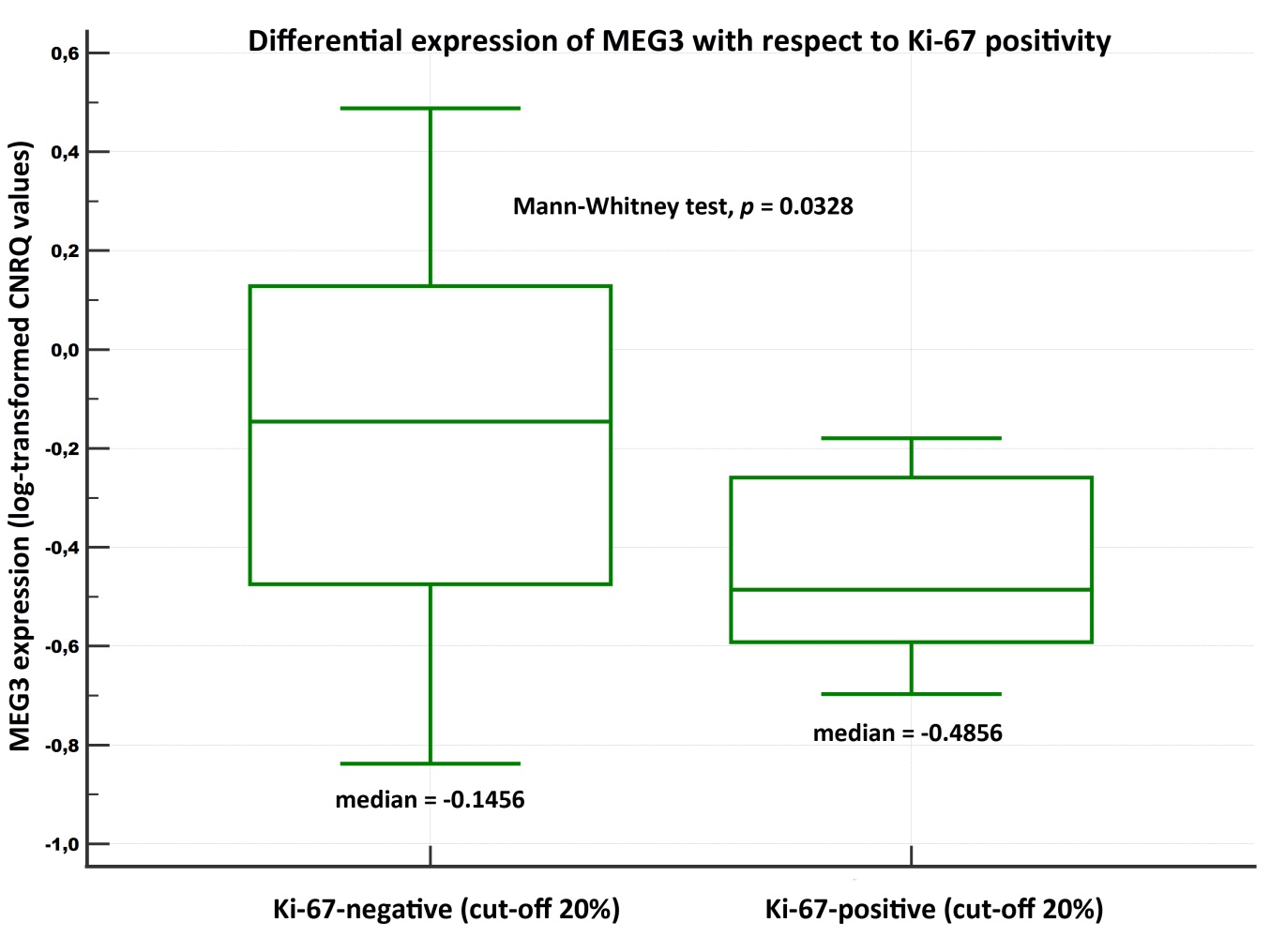
**

**Figure S2.** Relative expression of MEG3 in breast cancer tissue samples with respect to Ki-67 status (validation experiment)

Notes: A box of the box-plot is drawn from the 1st to 3rd quartile (the 25th and 75th percentiles). A horizontal line within a box plot represents the median. Horizontal lines are drawn at the highest value and the lowest expression value.

**
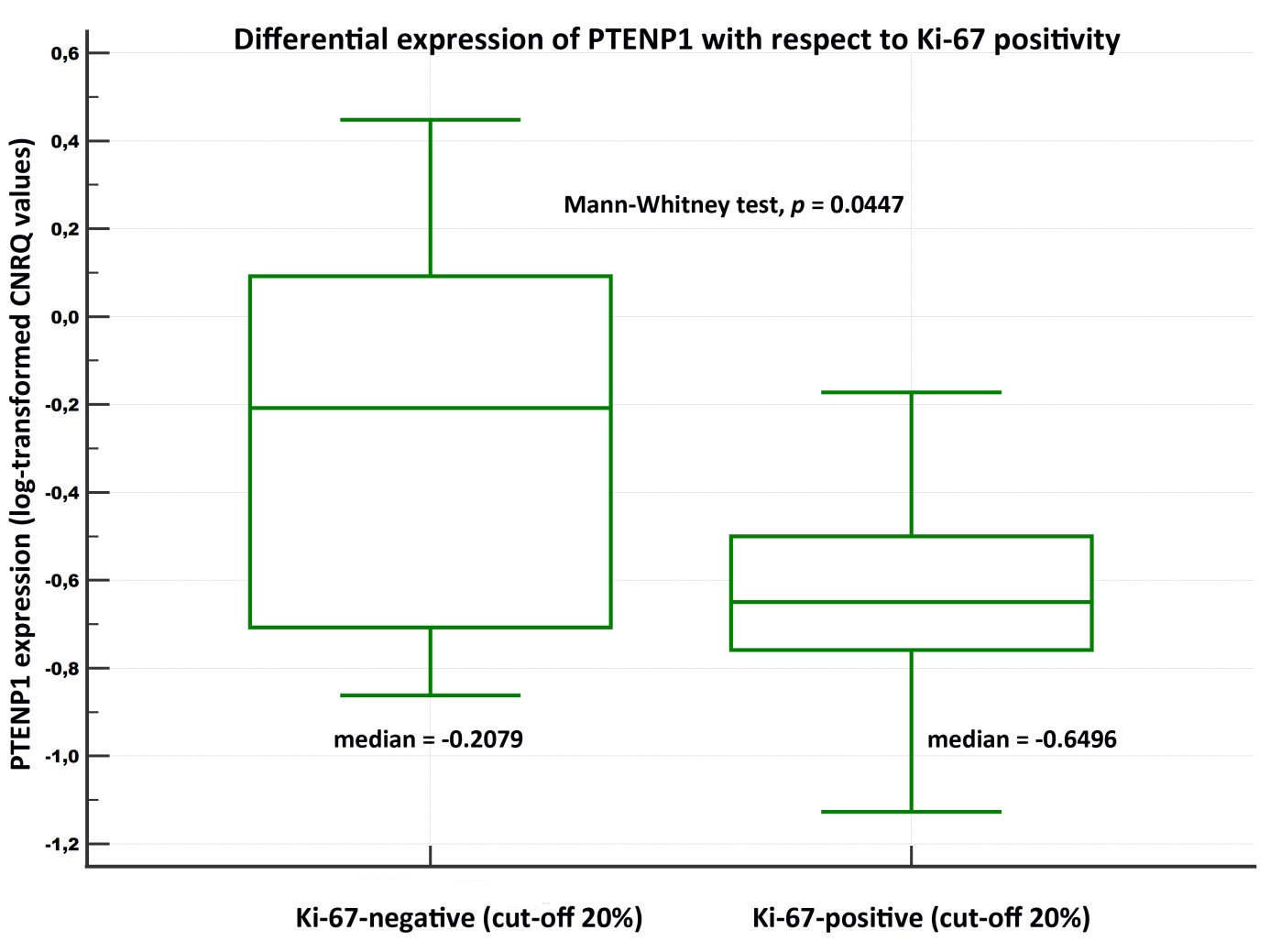
**

**Figure S3.** Relative expression of PTENP1 in breast cancer tissue samples with respect to Ki-67 status (validation experiment)

Notes: A box of the box-plot is drawn from the 1st to 3rd quartile (the 25th and 75th percentiles). A horizontal line within a box plot represents the median. Horizontal lines are drawn at the highest value and the lowest expression value.

**
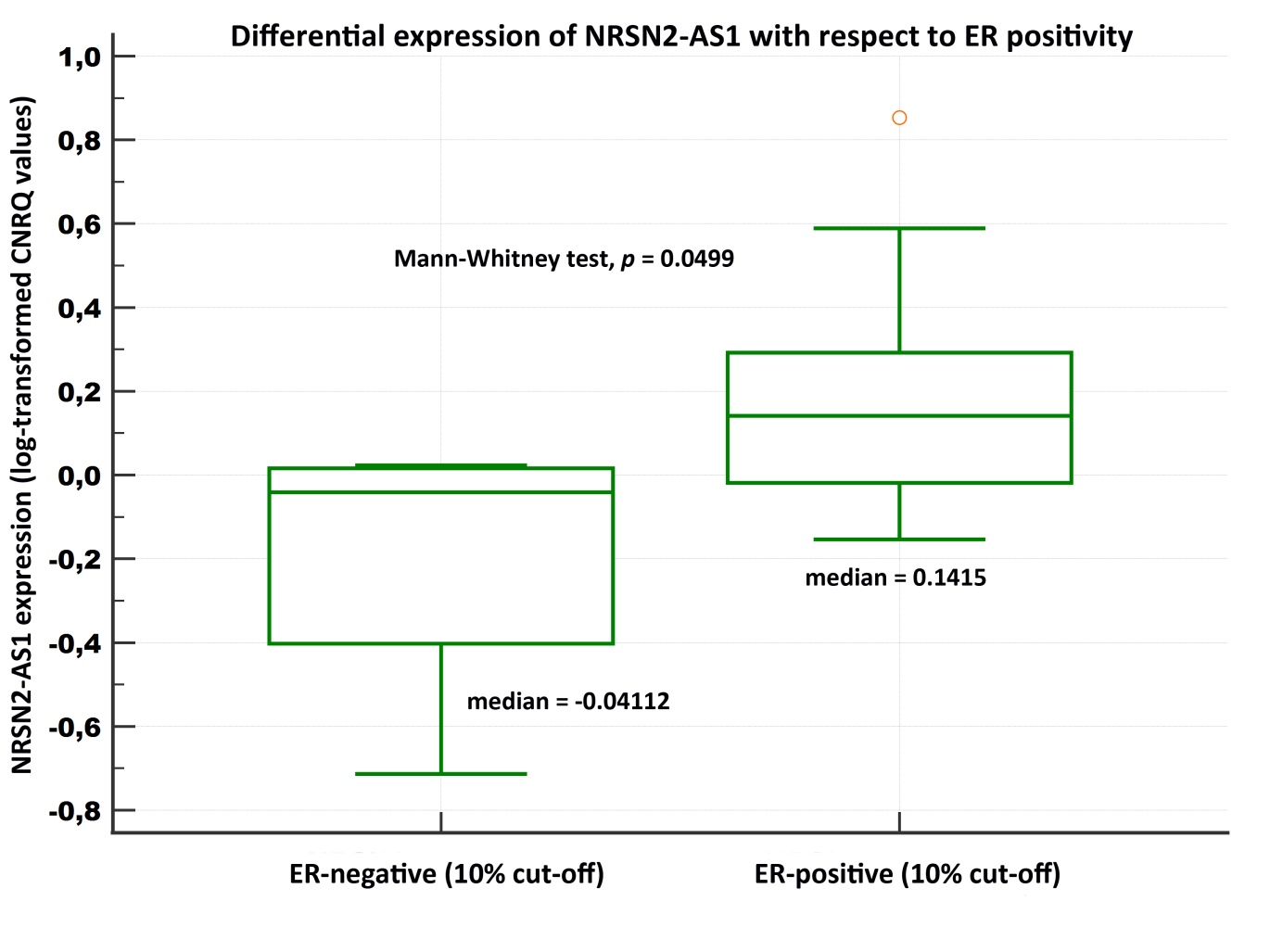
**

**Figure S4.** Relative expression of NRSN2-AS1 in breast cancer tissue samples with respect to ER status (validation experiment)

Notes: A box of the box-plot is drawn from the 1st to 3rd quartile (the 25th and 75th percentiles). A horizontal line within a box plot represents the median. Horizontal lines are drawn at the highest value and the lowest expression value. A circle represents an outside value.

**
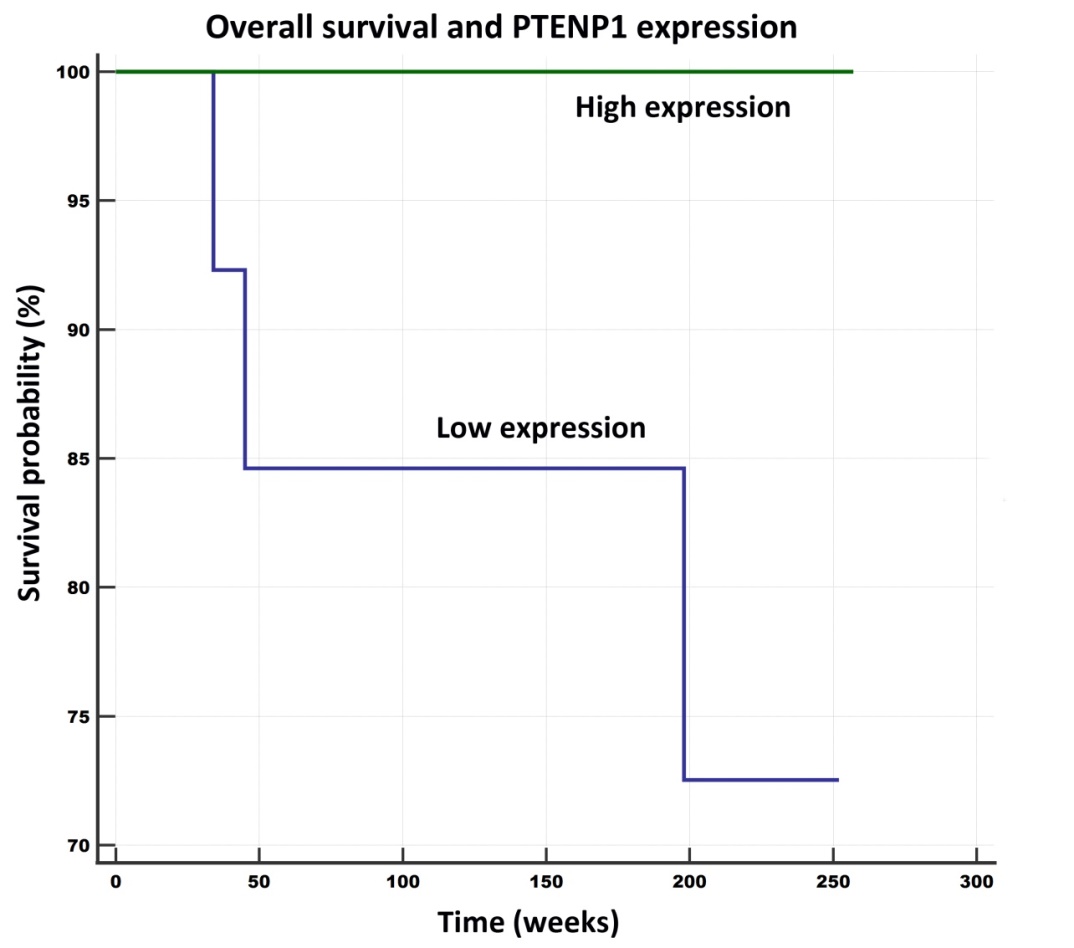
**

**Figure S5.** Overall survival in relation to PTENP1 expression levels

Notes: Univariate Kaplan-Meier survival curves for overall survival (OS) related to low and high concentrations of PTENP1 in breast cancer tumors. Mean OS for the low expression subgroup was 4.1 years (212.8 weeks), and for the high expression subgroup it was 4.9 years (257 weeks). Log-rank test, *p* = 0.0559 (marginally significant).

**
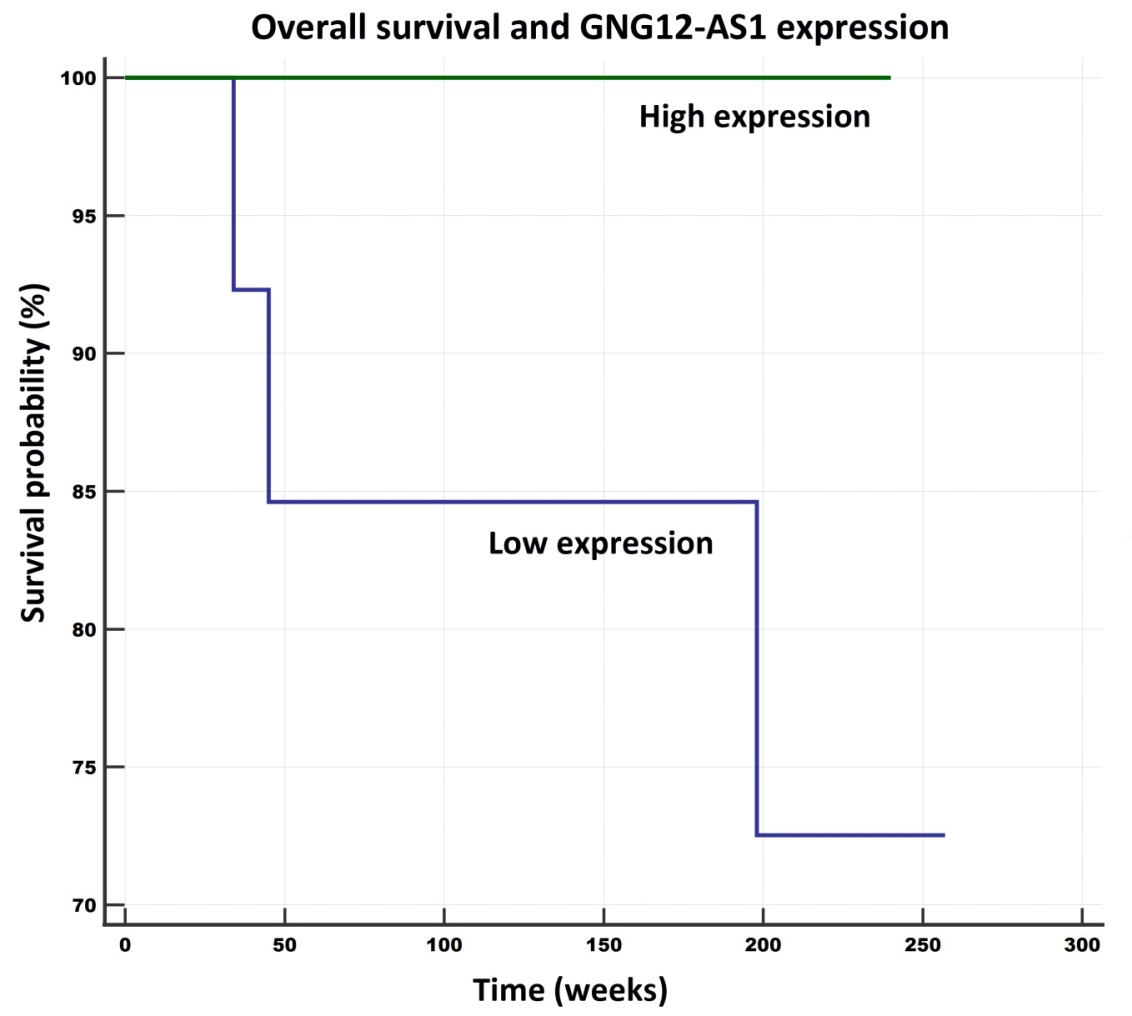
**

**Figure S6.** Overall survival in relation to GNG12-AS1 expression levels

Notes: Univariate Kaplan-Meier survival curves for overall survival (OS) related to low and high concentrations of GNG12-AS1 in breast cancer tumors. Mean OS for the low expression subgroup was 4.15 years (216.4 weeks), and for the high expression subgroup it was 4.6 years (240 weeks). Log-rank test, *p* = 0.0559 (marginally significant).


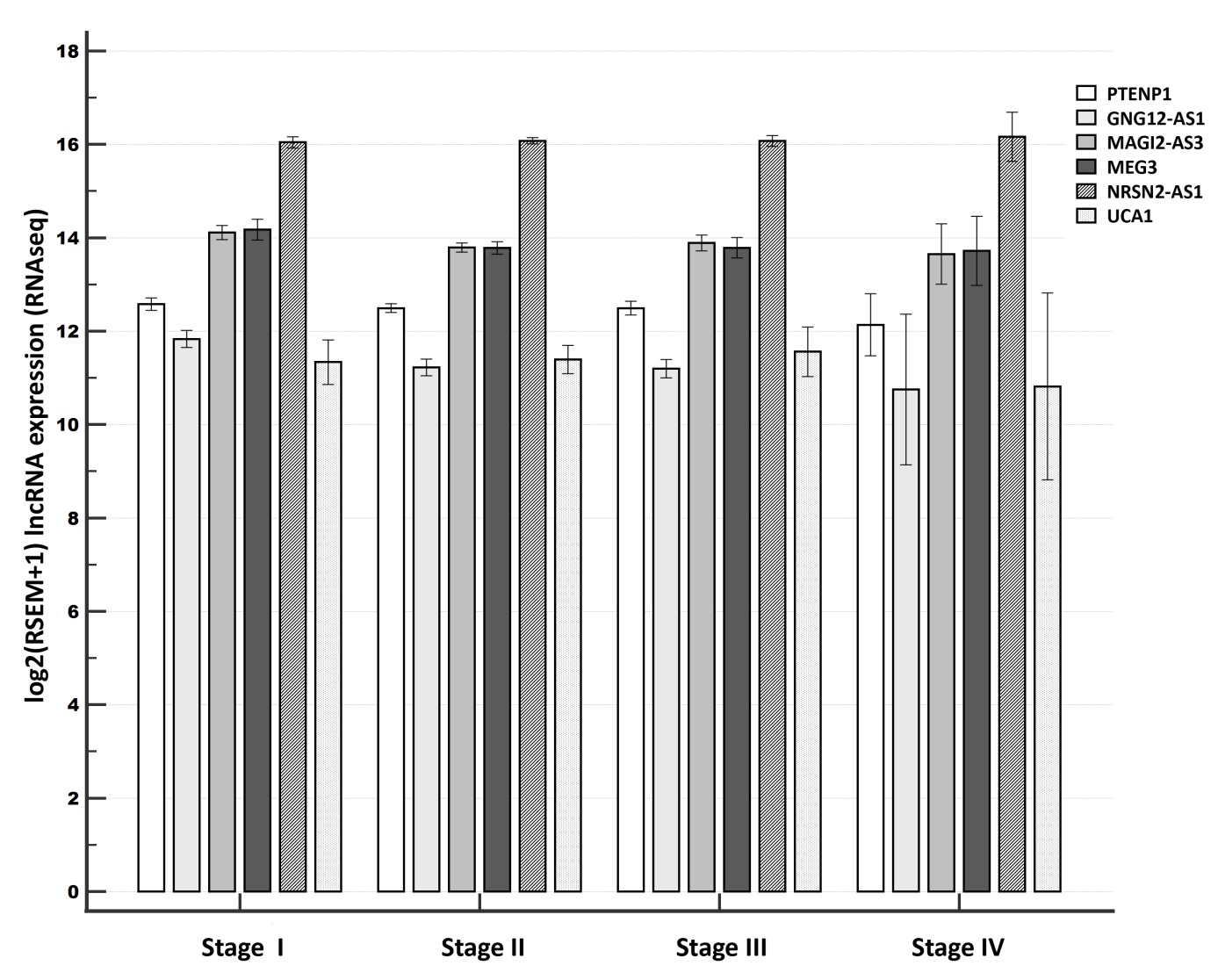


**Figure S7. Relative lncRNA expression across the different tumor stages (the GDC TCGA Breast Cancer (BRCA) dataset**

Notes: A clustered multiple-comparison graph showing mean lncRNA expression data (log-transformed CNRQ) as bar charts. Error bars indicate 95% CI for the mean.
